# Redefining Stability in Cultural Heritage Through Polymer Design: From Conservation Strategies to Plastic Degradation

**DOI:** 10.3390/polym18141783

**Published:** 2026-07-21

**Authors:** Elisabetta Ranucci, Jenny Alongi

**Affiliations:** Dipartimento di Chimica, Università degli Studi di Milano, Via C. Golgi 19, 20133 Milano, Italy; elisabetta.ranucci@unimi.it

**Keywords:** polymers for Cultural Heritage, functional polymers, adhesives, consolidants, coatings, polymeric gels, cellulose, wood, modern plastics

## Abstract

Polymers play a central and multifaceted role in cultural heritage science, serving both as functional materials in conservation treatments, such as cleaning, consolidation, adhesion and protection, and as constituents of a wide range of historical artefacts, including paper and canvas, waterlogged wooden wrecks, musical instruments, and modern plastics used in art. Although numerous studies have examined the use of polymers in archaeology and cultural heritage conservation, most have focused on specific polymers, individual conservation treatments, or categories of artefacts. A comprehensive and integrated assessment of the multifunctional role of polymers, both as conservation materials and as constituents of heritage objects, remains lacking. The aim of this review is to provide a critical and comprehensive overview of natural and synthetic polymers in cultural heritage science, examining their applications in conservation treatments, their long-term stability and aging, and the challenges and opportunities associated with their preservation and sustainable use. This review examines the main classes of natural and synthetic polymers used in conservation, evaluating their mechanisms of action, performance, limitations, and long-term behavior across different applications. It also examines the chemical decomposition pathways and the resulting degradation phenomena occurring in polymeric materials, both as conservation products and as constituents of cultural artefacts, together with current stabilization strategies aimed at mitigating aging and deterioration. This review provides a critical appraisal of current challenges and future perspectives in cultural heritage conservation, highlighting emerging trends and research directions for the development of more effective, sustainable, and compatible polymer-based solutions for cultural heritage conservation.

## Table of Contents

1. Introduction
*1.1. Conservation Workflow as a Framework for Polymer Selection and Application*
*1.2. Sustainability in Polymer-Based Conservation of Cultural Heritage: a Critical Synthesis of Principles and Constraints*
2. Functional Polymers in Conservation Science
*2.1. Functional Polymers for the Pre-Consolidation of Artefacts*
*2.2. Functional Polymers for the Cleaning of Artefacts*
    2.2.1. Hydrogels
    2.2.2. Organogels
    2.2.3. Semi-Interpenetrating (Semi-IPNs) and Interpenetrating Polymer Networks (IPNs)
    2.2.4. Stimuli-Responsive Hydrogels
    2.2.5. Polymeric Cleaning Systems in Cultural Heritage: A Critical Synthesis and Future Outlook
*2.3. Functional Polymers for the Consolidation of Artefacts*
*2.4. Functional Polymers as Adhesives in Cultural Heritage Conservation*
*2.5. Functional Polymers for the Surface Protection of Artefacts*
    2.5.1. Stone Surfaces: Case Studies
    2.5.2. Metals (Bronze, Copper Alloys, Iron): Case Studies
    2.5.3. Polychrome and Painted Surfaces: Case Studies
    2.5.4. Ceramics and Terracotta: Case Studies
    2.5.5. Wooden Objects: Case Studies
    2.5.6. Polymer Coatings in Cultural Heritage: A Comparative Synthesis and Critical Assessment
3. Polymers as Constituent Materials in Artefacts
*3.1. Natural Polymers*
    3.1.1. Cellulose-Based Artefacts
    3.1.2. Paper: Case Studies
    3.1.3. Wood-Based Artefacts: Case Studies
    3.1.4. Wooden Musical Instruments: Case Studies
*3.2. Synthetic Polymers in Cultural Heritage*
    3.2.1. Historical Development of Plastics in Art and Design
    3.2.2. Plastics in Art and Design: Materials, Properties, and Conservation
    3.2.3. Polymer-Based Cultural Heritage: A Critical Analysis of the Current State of Art in the Conservation of Plastics in Art and Future Perspectives
4. Conclusions and Future Perspectives

## 1. Introduction

Polymers play a central and multifaceted role in cultural heritage conservation, acting both as functional materials used in restoration—e.g., cleaning, consolidation, [1] and protection [2,3]—and as constituents of historical artefacts. They are found not only in traditional substrates such as paper, canvas, and wood (e.g., shipwreck, musical instruments), but also in modern materials, including synthetic polymers widely used in 20th-century and contemporary art [4]. Recognizing this dual role is essential for developing effective, sustainable, and scientifically grounded conservation strategies.

In conservation practice, polymers are designed to meet specific functional requirements by tuning their physicochemical properties [5,6]. While traditional interventions relied mainly on natural polymers (e.g., proteins, polysaccharides, and terpenoid resins used as binders, adhesives, or coatings), advances in polymer chemistry and colloid science have introduced more sophisticated systems, such as gels, microemulsions, and nanostructured materials. These systems enable more selective, controlled, and less invasive treatments and more predictable aging behavior, in line with current conservation principles of compatibility, stability, and reversibility.

At the same time, polymers themselves constitute a wide range of artworks in cultural heritage. Natural polymeric substrates, such as cellulose in paper, lignocellulosic matrices in wooden artefacts, and protein-based media in paint layers or adhesives are intrinsically sensitive to environmental fluctuations. Their chemical heterogeneity, when exposed to oxidative, hydrolytic, or biological degradation processes, gives rise to multifactorial aging pathways whose complexity requires advanced analytical methodologies and highly specialized diagnostic frameworks.

The developing of synthetic polymers throughout the 20th and 21st centuries has further compounded this material complexity, introducing it into cultural heritage collections objects. Acrylic paints, polyurethane foams (PUFs), polyvinyl chloride (PVC)-based artefacts, and an ever-expanding array of plastic materials constitute a category of artworks whose aging processes manifest in phenomena such as plasticizer migration, embrittlement, chromatic alteration, and phase separation. As a result, polymer-based heritage presents significant conservation challenges. Addressing them requires interdisciplinary approaches that combine polymer science, materials analysis, and conservation theory with the aim of both understanding degradation mechanisms and developing appropriate preservation strategies.

Building on these considerations, this review presents a critical synthesis of the roles, limitations, and future directions of polymers in cultural heritage, encompassing both their use as conservation materials and their presence as artefacts.

### 1.1. Conservation Workflow as a Framework for Polymer Selection and Application

Recognizing polymers both as active conservation materials and as vulnerable components of artworks allows conservators to assess risks, anticipate degradation, and plan interventions that balance immediate treatment needs with long-term stability. This perspective supports the integration of polymer science across the main phases of the conservation process, promoting compatible, reversible, and preventive approaches [7]:

○ Anamnesis: identification of the artefact, its history, and environmental context.
○ Diagnosis: characterization of materials, degradation phenomena, and conservation needs.
○ Pre-consolidation: stabilization of severely deteriorated materials.
○ Cleaning: removal of surface contaminants (e.g., particulate, dirt or grime) and altered layers.
Consolidation: reinforcement of weakened structures.
○ Surface protection: application of coatings to shield the artefact from environmental agents.
○ Maintenance: periodic monitoring and reassessment of the conservation state.

Polymers play a key role throughout these stages. Their identification in the anamnesis phase informs risk assessment and preventive strategies, while analytical techniques such as FT-IR (Fourier Transform Infrared), Raman, and NMR (Nuclear Magnetic Resonance) spectroscopies enable material characterization and guide treatment selection during diagnosis. In pre-consolidation, low-viscosity polymers and nanostructured systems provide temporary stabilization of fragile substrates. In cleaning, advanced polymer-based systems (e.g., micelles [8,9], emulsions, microemulsions [10,11], gels [12,13,14,15,16], and semi-interpenetrating networks (semi-IPNs) [17,18] allow selective removal of contaminants while limiting solvent penetration and mechanical stress. During consolidation, polymeric materials such as acrylics, poly(vinyl acetate) (PVAC), polyvinyl alcohol (PVA), siloxanes, and hybrid systems restore cohesion in degraded substrates, with their performance governed by molecular design. For surface protection, polymer coatings provide barriers against moisture, UV radiation, and pollutants while maintaining optical compatibility and reversibility. Finally, in maintenance, monitoring changes in the chemical (molecular structure, molecular weight) and physical properties of polymer-based treatments (e.g., color, gloss, elasticity, etc.) supports informed decisions on re-treatment and long-term conservation strategies.

### 1.2. Sustainability in Polymer-Based Conservation of Cultural Heritage: A Critical Synthesis of Principles and Constraints

In the context of the United Nations 2030 Agenda and UNESCO (United nations educational, scientific and cultural organization) [19,20] frameworks, cultural heritage conservation carries a shared responsibility to align preservation strategies with environmental and social sustainability objectives [21]. Within these frameworks, cultural heritage encompasses not only artefacts and museum collections, but also natural heritage, including landscapes, ecosystems, and biodiversity, thereby reinforcing the need for approaches that consider both cultural and environmental systems.

This requires materials and methodologies capable of reconciling conservation performance with responsible resource use, positioning polymers as key enablers in translating sustainability goals into practice-oriented solutions. In line with these principles, the Sustainable Development Goals (SDGs) [22] provide a global reference for integrating environmental responsibility, social inclusion, and economic sustainability across scientific and cultural domains.

In conservation practice, polymer-based materials exemplify the intersection of heritage preservation and sustainability: modern polymeric systems enable minimally invasive, reversible, and controlled treatments, reducing hazardous chemical use (SDG 12) and lowering energy consumption and emissions (SDG 13). Advances in bio-based, recyclable, and biodegradable polymers further support the integration of circular-economy principles into conservation practice.

**Figure 1:**
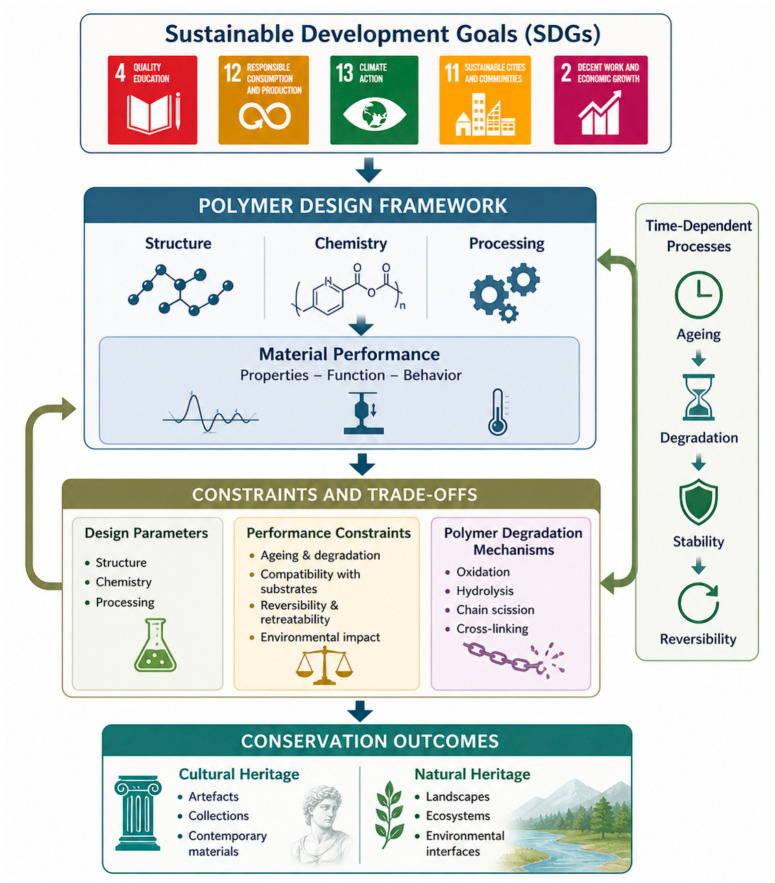
Conceptual framework linking sustainable polymer design with conservation science. Polymer structure, chemistry, and processing determine material performance, while design constraints and degradation mechanisms govern long-term stability and reversibility. Time-dependent aging informs iterative redesign strategies, ultimately supporting the conservation of cultural and natural heritage. Blue arrows indicate the main design pathway, whereas green arrows represent feedback loops associated with aging and degradation. Panel colors distinguish the main conceptual domains: polymer design (blue), constraints and trade-offs (beige), and conservation outcomes (green).

Functionally, polymers stabilize fragile artefacts, reinforce deteriorated substrates, and provide protective coatings with tunable chemical and mechanical properties, ensuring long-term preservation with minimal environmental impact (SDG 11.4). Beyond treatment, the application of polymer science in museums also contributes to education [23] (SDG 4), inclusive access and broader social engagement (SDG 5 and SDG 10), sustainable cultural and economic activity (SDG 8), and peaceful, equitable societies (SDG 16) (Figure 1).

Taken together, these aspects highlight the need for a more critically informed integration of sustainability principles into polymer development, where material innovation is evaluated not only in terms of performance, but also in relation to its long-term environmental and cultural impact.

Within this perspective, the assessment of polymer systems increasingly requires consideration of their broader cultural and environmental implications.

## 2. Functional Polymers in Conservation Science

Functional polymers, designed to perform specific tasks, are essential in conservation science. Their tunable properties allow conservators to support all stages of treatment, from cleaning and stabilizing fragile surfaces to consolidating deteriorated materials and applying long-lasting protective coatings. Their versatility enables minimally invasive, reversible, and sustainable interventions, making polymers key tools in the preservation of cultural heritage [24,25].

### 2.1. Functional Polymers for the Pre-Consolidation of Artefacts

Pre-consolidation is a preparatory step in conservation aimed at stabilizing fragile, friable or powdery surfaces before cleaning or consolidation interventions. Materials such as deteriorated stone, plaster, mural pigments, or deteriorated wood can be easily damaged by solvents or mechanical tools [24]. In the absence of pre-stabilization, subsequent treatments such as cleaning or consolidation can inadvertently cause pigment loss, detachment of surface layers, or accelerated material decay.

Traditionally, pre-consolidation has been carried out using low-viscosity natural or synthetic resins dissolved in alcohol or organic solvents [24]. Although effective in stabilizing delicate surfaces, they have significant drawbacks: solvent penetration may disturb underlying layers, and resin films can become uneven or excessively rigid, potentially compromising the effectiveness of subsequent consolidation treatments. Solvent-based treatments also pose environmental and health risks due to volatile organic compound (VOC) emissions. To address these limitations, a range of polymers has been developed with tailored properties, including controlled viscosity, low invasiveness, reversibility, and compatibility with subsequent conservation treatments (Table 1). Specifically, water-based polymer systems, such as PVAC [26], significantly reduce the use of organic solvents and associated VOC emissions, thereby enhancing environmental sustainability. Similarly, biopolymers, including cellulose derivatives [27], represent renewable and biodegradable alternatives that further mitigate long-term environmental impact.

### 2.2. Functional Polymers for the Cleaning of Artefacts

The cleaning of artefacts is one of the most delicate and critical phases of conservation, as the removal of unwanted deposits such as dirt/soil, soot, surface crusts, aged varnishes, coatings, or adhesives, overpainted or degraded layers, and traces of smearing or vandalism, including graffiti, must be carried out without altering or damaging the underlying materials [24]. Traditional cleaning approaches, such as aqueous systems, organic solvents, and mechanical methods, have long been valued for their effectiveness and ease of use [29]. However, as previously discussed, they present significant limitations: solvents may penetrate porous substrates or solubilize original materials [30], while mechanical methods can be abrasive or lack selectivity, in addition to raising toxicity and environmental concerns. In easel or wall paintings, these issues become even more critical and analytically complex, as solvent/pigment interactions at the molecular level can induce subtle yet significant chemical and physical alterations, complicating both diagnostic assessment and conservation decision-making [30,31].

In recent decades, functional polymers have emerged as advanced tools capable of addressing many of these issues [5,6]. By confining liquids within a polymer matrix, they enable highly selective and controlled cleaning, reducing solvent release and limiting penetration into the artwork. Among the systems currently under investigation are gels (hydrogels and organogels), semi-IPNs and interpenetrating polymer networks, stimuli-responsive networks, and polymer-stabilized emulsions, all offering safer, more precise, and more sustainable cleaning strategies [8,9,10,11,12,13,14,15].

In conservation science, gels are broadly classified as physical or chemical: the former consist of networks held together by reversible non-covalent interactions (e.g., hydrogen bonds or electrostatic forces), while the latter are three-dimensional (3D) covalently crosslinked networks structures that provide greater structural stability.

In all these polymeric systems, solvent confinement is achieved through [18,32]:

− Network entrapment (physical confinement): the gel mesh (pore size and tortuosity) traps solvent, micelles, or nanodroplets, regulating their mobility.
− Capillary and interfacial effects: capillary forces, interfacial tension, and wetting stabilize confined fluids and limit spreading and evaporation.
− Viscoelastic damping: gel viscosity suppresses convection, promoting diffusion-controlled transport and reducing solvent penetration.
− Affinity and selective partitioning (chemical confinement): polymer/solvent interactions (e.g., hydrogen bonding, polarity matching, van der Waals forces) govern uptake, retention, and release.
− Micro-/nano-emulsion confinement: when present, droplets embedded in the network are controlled by size, surfactant shell, and gel interactions, reducing free solvent activity.
− Porosity control: porous organogels can absorb excess solvent and act as reservoirs, wicking it away from the interface after treatment [33].

#### 2.2.1. Hydrogels

**Figure 2:**
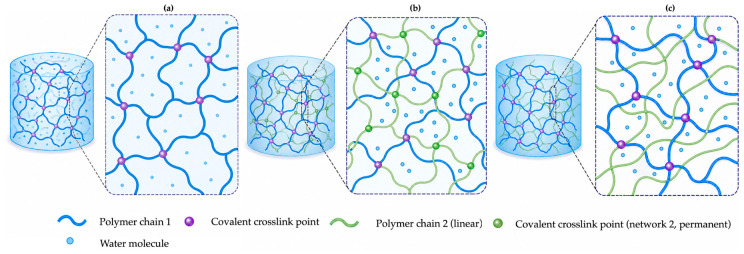
Schematic of a crosslinked polymer network (**a**), an IPN (**b**) and a semi-IPN (**c**).

Hydrogels are extensively applied in conservation science for the cleaning of water-sensitive and porous substrates. They consist of highly hydrophilic, three-dimensional crosslinked polymer networks capable of retaining substantial amounts of water while maintaining structural integrity (Figure 2a) [18,32,34,35]. This feature enables their use as carriers for cleaning systems, such as surfactants, pH-adjusted solutions, and chelating agents, ensuring controlled and localized release. Modulation of crosslink density, swelling behavior, and polymer/substrate interactions allows precise tuning of solvent delivery, residence time, and mechanical action. As a result, hydrogels promote the selective removal of surface deposits, aged varnishes, and particulate matter, while minimizing solvent overexposure, leaching, and substrate penetration. Their viscoelastic properties further facilitate safe application on vertical, irregular, and fragile surfaces, reducing mechanical stress and abrasion [32,34].

In conservation science, hydrogels commonly employed for cleaning applications can be broadly classified into polysaccharide-based (natural) and synthetic systems (Table 2). Among natural hydrogels, those derived from polysaccharides are typically classified as physical gels and have been widely employed in the treatment of water-sensitive and delicate substrates, such as paper and parchment [36]. Agarose-, agar-, and Gellan gum-based gels are the most established materials in this context.

**Figure 3:**
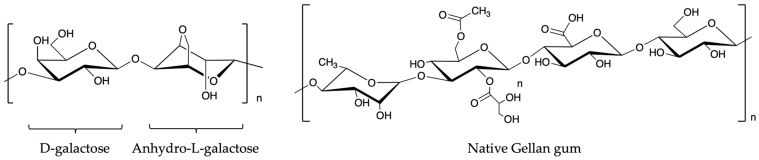
Repeating units of agarose (poly[(1→3)-β-D-galactopyranose-(1→4)-3,6-anhydro-α-L-galactopyranose]) and Gellan gum (poly[(1→3)-β-D-glucopyranosyl-(1→4)-β-D-glucuronopyranosyl-(1→4)-β-D-glucopyranosyl-(1→4)-α-L-rhamnopyranosyl]).

Agarose is a linear polysaccharide (molecular weight ~120,000) extracted from red seaweeds, mainly *Gelidiales and Gracilariales* [37], and composed of repeating agarobiose units (Figure 3) that form thermo-reversible hydrogels upon cooling from hot aqueous solutions [34,38].

**Figure 4:**
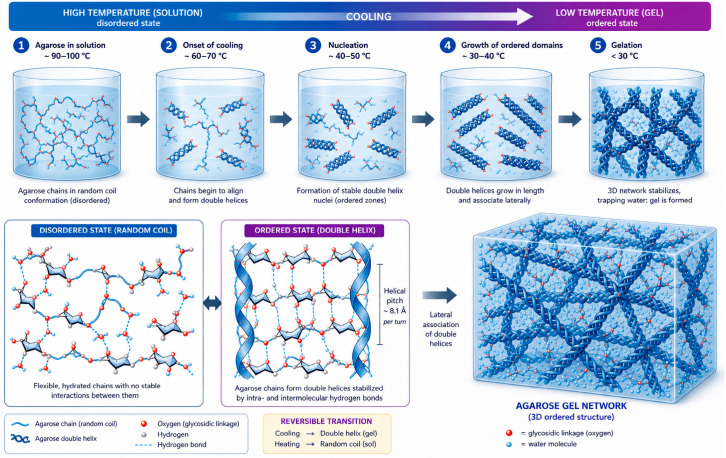
Schematic illustration of the thermoreversible gelation of agarose. Cooling induces the transition from disordered random coils to ordered double helices, followed by their lateral association into a three-dimensional network that entraps water and forms a gel. Heating reverses the process, restoring the sol state. Blue arrows indicate the sequential stages of gelation, whereas the double-headed arrow highlights the reversible sol–gel transition.

The gel–sol transition of agarose has been extensively investigated due to its relevance in both fundamental polymer physics and practical applications. Below the gelation temperature (T_gel_), agarose chains undergo a conformational transition from random coils to more ordered structures, assembling into double helices stabilized primarily by hydrogen bonding (Figure 3 and Figure 4) [39]. These helices further aggregate into supramolecular aggregates that act as junction zones, giving rise to a three-dimensional network with water-filled channels. At low concentrations, agarose gels are soft, while at high concentrations they become more rigid [40]. The gelation process is thermo-reversible: heating above T_gel_ disrupts the intermolecular interactions and restores the sol state. This well-defined coil–helix transition, together with the hierarchical organization of helical aggregates, makes agarose a model system for understanding the structure–property relationships in physically crosslinked hydrogels. Agarose gels provide stable, porous networks with good mechanical strength and controlled water release, enabling localized cleaning while limiting swelling in hygroscopic substrates, as for instance, in paper [41,42].

Agar consists primarily of a mixture of agarose and agaropectin, also derived from red seaweeds [37]. Similarly thermo-reversible and easy to prepare, agar gels offer tunable rigidity, porosity, and water retention through adjustments in concentration and composition. Agar gels have been widely applied for cleaning and poulticing treatments, ensuring controlled moisture delivery with minimal mechanical stress [43].

Gellan gum is an anionic microbial polysaccharide produced by fermentation, typically by *Sphingomonas elodea*, composed of repeating tetrasaccharide units that forms gels at low concentrations (Figure 3) [44]. Its properties can be modulated, from rigid and brittle to soft and elastic, depending on acyl content and cation presence. Owing to their transparency, chemical stability, and controlled release behavior, Gellan gum gels have gained increasing relevance in conservation practice, particularly for paper treatments [45,46,47,48].

Table 2 summarizes the most relevant applications of polysaccharide-based hydrogels across different types of artworks, highlighting their main advantages and limitations.

Polysaccharide-based gels are inherently limited by residue deposition on treated substrates. Additionally, naturally derived hydrogels require extemporaneous preparation and display pronounced syneresis, thereby constraining their use on highly sensitive materials such as paper. These shortcomings have driven the development of more stable, non-perishable systems with enhanced mechanical robustness and solvent retention capacity. In this context, synthetic hydrogels afford superior control over physicochemical properties, as well as improved selectivity and reproducibility, supporting their application across a wide range of substrates, including paper, canvas, painted surfaces, wall paintings, stone, and modern and contemporary artworks [49].

Among the earliest synthetic gel systems introduced in conservation practice, physically crosslinked PVA hydrogels have been extensively studied. The earliest PVA-based systems were obtained by cast-drying (peelable systems), or freeze–thawing (physical cryogels) [12,50], where gelation was driven by phase separation and crystalline domains acting as reversible junctions [51,52].

These semicrystalline networks provide high viscoelasticity, mechanical integrity, and effective solvent retention.

**Figure 5:**
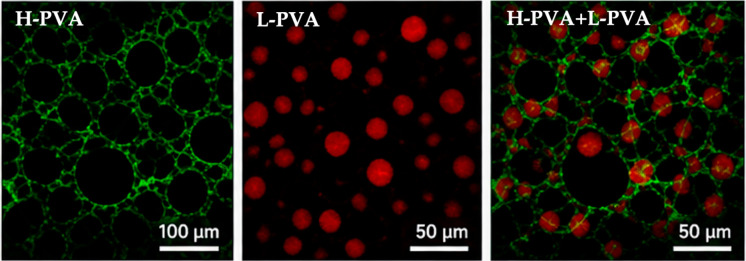
Cartoon representing the high-molecular-weight (H-PVA)/low-molecular-weight (L-PVA) liquid–liquid phase distribution in a “twin-chain” PVA-based hydrogel after freeze–thawing cycles.

A recently introduced class of soft materials, referred to as “twin-chain” polymer networks, has significantly advanced the design of functional hydrogels for the cleaning of artefacts, with many reported systems based on PVA [12,53]. In these systems, phase separation between PVA fractions with different molecular weights leads to the formation of low-molecular-weight PVA-rich domains dispersed within a continuous high-molecular-weight matrix. Following freeze–thaw-induced physical crosslinking, the more soluble low-molecular-weight fraction can be selectively leached out during aqueous washing, thereby generating an interconnected porous structure (Figure 5). The resulting network combines structural stability with efficient fluid uptake and controlled solvent release. This straightforward, low-energy process yields sponge-like architectures characterized by highly interconnected porosity and tunable tortuosity. The resulting networks exhibit unique transport and retention properties, which translate into exceptional performance in applications requiring controlled fluid delivery. These systems have enabled safe and time-efficient cleaning of highly sensitive surfaces, such as for instance paintings by Jackson Pollock, Roy Lichtenstein, and others [53] and Pablo Picasso [54].

**Figure 6:**
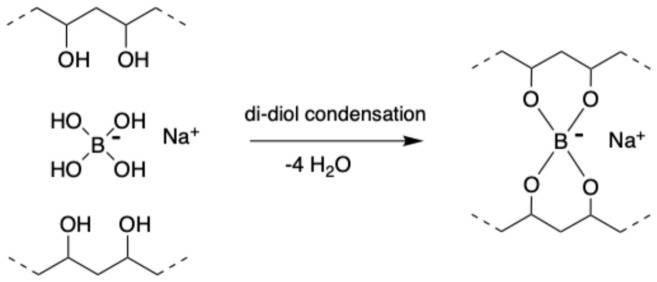
Synthesis of the PVA–borax reversible hydrogel.

PVA–borax formulations also represent a significant advancement in controlled cleaning methodologies. They rely on the reversible physical crosslinking of PVA chains via borate ions (Figure 6) [55], forming viscoelastic networks with high surface adaptability and straightforward post-treatment removal [56,57]. PVA–borax hydrogels have been widely employed for the removal of synthetic coatings, soil, and degradation products from a variety of substrates, including wall paintings, stone, and textiles, demonstrating high cleaning efficacy and minimal mechanical impact [58,59]. However, their intrinsic physical crosslinking mechanism also entails certain limitations, such as susceptibility to syneresis, limited long-term stability, and the potential release of residues under specific conditions [60]. These drawbacks have stimulated further research toward more stable and retentive gel systems, including covalently crosslinked and double-network hydrogels (DN, Section 2.2.3), marking an important evolution in conservation cleaning strategies [12,34,57].

Advanced cleaning systems based on chemically crosslinked PVA hydrogels were developed from mixtures of PVA and telechelic PVA (PVA-t). The latter was obtained via periodate oxidation of vicinal diols located in head-to-head sequences along the PVA backbone, resulting in selective chain cleavage and the formation of terminal aldehyde groups. PVA bearing terminal aldehyde groups (PVA-t) acts as a macromolecular crosslinker, forming stable networks via acetal linkages. PVA/PVA-t hydrogel systems exhibit enhanced mechanical stability, solvent retention, and compatibility with cellulose-based substrates, making them particularly suitable for the controlled cleaning of paper artworks [61,62].

Alongside PVA-based systems, polyacrylamide (PAAm) gels have emerged as a versatile class of synthetic hydrogels in conservation. They are typically prepared by free-radical polymerization of acrylamide in water, in the presence of a bifunctional crosslinker (typically N,N′-methylenebisacrylamide). Initiation can be achieved through redox, thermal, or photochemical systems. This synthetic flexibility enables fine control over network architecture, porosity, and mechanical properties, supporting their application in highly controlled and selective cleaning protocols, offering greater reproducibility and predictable behavior compared to natural gels. PAAm hydrogels have been successfully applied to the cleaning of wall paintings, stone, and painted surfaces, where they provide effective removal of contaminants while ensuring minimal residue and a high reproducibility of treatment [12,63].

**Figure 7:**
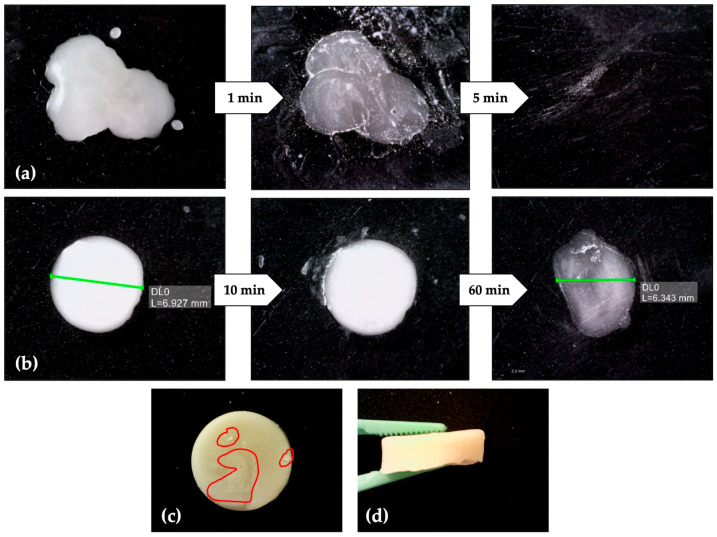
Wax cleaning test: cleaned surface using ethanol-soaked cotton swab (**a**), and H-M-GLY/MMT swollen in a 5% aqueous ethanol solution (**b**); H-M-GLY/MMT after wax cleaning (**c**,**d**). In (**c**), the red lines indicate the presence of wax traces.

Polyamidoamine (PAA) hydrogels have recently emerged as a versatile and highly tunable platform in paper conservation, marking a significant step forward in the design of functional cleaning systems [64]. PAA hydrogel samples have been synthesized via aza-Michael polyaddition of N,N′-methylenebisacrylamide and glycine to yield acrylamide-terminated PAA oligomers, followed by redox-initiated crosslinking into three-dimensional networks. Precise control over synthesis parameters, including pH, concentration, and terminal functionality, enables fine modulation of mechanical properties, swelling behavior, and acid–base characteristics, ultimately dictating their interaction with sensitive cellulosic substrates. The incorporation of montmorillonite as a nanofiller further extends this design space: when dispersed in the precursor solution under acidic conditions, strong electrostatic interactions between cationic PAA chains and negatively charged clay layers promote intercalation and homogeneous distribution, yielding nanocomposite hydrogels with enhanced mechanical robustness and dimensional stability. Functionally, PAA-based systems, both pristine and clay-reinforced, when swollen in hydroalcoholic mixtures, exhibited high selectivity in the removal of wax deposits and other surface contaminants, while preserving inks and maintaining the mechanical integrity of treated artefacts (Figure 7). Notably, beyond their cleaning performance, these hydrogels contributed to surface deacidification, addressing a critical factor in the long-term stabilization of aged paper.

**Table 2:** Application of hydrogels in the cleaning of cultural heritage artworks.

Hydrogel	Gel Type	Artwork to Be Cleaned	Result	Advantages	Drawbacks	Reference
Polysaccharide-based hydrogels						
Agarose	Physical rigid gel	- Paper - Painted surface - Fresco mock-ups	Removal of: - dirt - grime - adhesives - aged consolidants	- Easy to prepare - Inexpensive - Good water control - Peelable with minimal residue	- Limited solvent compatibility - Too high rigidity	[43]
Agar-based solvent-gels	Physical rigid gel	Paper	Removal of apolar residues	Excellent solvent confinement	-	[65]
Agar + citrus pectin	Physical gel	Oil paintings	Removal of: - dirt - adhesive residues	Simple and reproducible method	-	[66]
Gellan gum	Physical rigid gel	- Paper - Parchment	Removal of: - adhesives - stains	- Good solvent retention - Minimal diffusion	It must be tailored to avoid over-wetting	[47]
Gellan gum	Physical gel	- 18th-century prints - Modern papers	Removal of: - cellulose degradation products - water-soluble soiling	- Fast action - Low penetration - Tunable particle size	- Complex preparation - Handling requires care	[48]
Functionalized Gellan gum with EDTA ^(a)^	Physical gel	Metal objects	Removal of complex metallic stains	Tunable gel formulation	An in-depth study on the formulation is required	[67]
Synthetic hydrogels						
PVA ^(b)^	Chemical gel	- Paper - Painted surfaces	Removal of: - dirt - water-soluble contaminants	- Tunable mechanical properties - Good water retainment - Biocompatible	Careful preparation	[61]
PVA ^(b)^ + sodium tetraborate ^(c)^	Chemical gel	- Paper - Canvas - Wall paintings	Removal of: - grime - starch residues	- Strong peelable gels - Minimal residue	Borate incompatibility with acidic papers	[68]
pHEMA ^(d)^/PVP ^(e)^	Chemical gel	- Paper - Manuscripts	Removal of: - dirt - aged residues	- Highly controllable release of solvents - No residues	- Complex synthesis - It requires precise formulation	[69,70]
PAAm ^(f)^	Chemical gel	- Paper - Canvas - Painted surfaces	Controlled release of solvents	- Customizable crosslinking - Mechanically stable - Widely studied	- Acrylamide monomer is toxic - Careful handling required - Potential residues if not washed	[71]
Nanorestore gel^® (g)^: PVA + Ca(OH)_2_ or CaO [72]	Chemical gel	- Oil paintings - Acrylic paintings - Contemporary art	Removal of: - oily grime - aged varnish	- High selectivity - Low mechanical stress - Minimal residues	More complex and expensive synthesis	[73]
Nanorestore gel^®^ Peggy ^(g)^ [72]	Chemical gel	- Acrylic paintings - Modern art surfaces	Removal of grime without swelling acrylic paint	Designed for modern materials, safe on sensitive polymers	Limited to specific solvents compatible with PEG system	[74,75]
PAA ^(h)^	Chemical gel	- Paper artefacts - Modern art surfaces	- Selective cleaning - Removal of polar contaminants	- Highly tunable chemistry - Good swelling control - Low leaving residues - Mild action	-	[64]

^(a)^ Ethylenediaminetetraacetic acid. ^(b)^ Poly(vinyl alcohol). ^(c)^ Borax. ^(d)^ Poly(hydroxyethyl methacrylate). ^(e)^ Poly(vinyl pyrrolidone). ^(f)^ Polyacrylamide. ^(g)^ Crosslinked poly(vinyl alcohol). ^(h)^ Polyamidoamine.

#### 2.2.2. Organogels

Organogels have emerged as effective polymeric systems for the cleaning of surfaces contaminated with hydrophobic or solvent-soluble deposits, such as aged varnishes, natural resins, and greasy materials. These viscoelastic networks immobilize organic solvents within their structure, enabling controlled solvent release and minimizing direct interaction with the substrate [33,76,77]. Similarly to hydrogels, organogels limit direct interaction with the substrate and reduce undesired chemical alterations and can be applied on vertical or irregular surfaces.

Additionally, PVA–borax/agarose double network hydrogels have been successfully extended to hydro/cosolvent systems, where water–alcohol mixtures (e.g., up to ~20–25 wt% propanol) are stably confined within the polymer network, significantly enhancing solubilization power while preserving controlled release and mechanical integrity [58]. These “hydro/cosolvent gels”, have demonstrated remarkable effectiveness in the removal of hydrophobic contaminants such as aged varnishes from artworks. Table 3 lists the main organogels for cleaning artworks in cultural heritage.

#### 2.2.3. Semi-Interpenetrating (Semi-IPNs) and Interpenetrating Polymer Networks (IPNs)

Semi-IPNs are hybrid systems comprising at least two components: a permanently crosslinked polymer network and a second, typically linear or branched polymer physically entangled within it without covalent bonds (Figure 2b) [86]. This architecture decouples mechanical and transport properties, enabling independent tuning of elasticity, porosity, and solvent diffusion. The crosslinked network provides structural integrity and mechanical strength, while the interlaced chains modulate mesh size and define mass transport pathways, thereby controlling swelling, diffusion, and interfacial interactions. Their mobility is crucial for regulating solvent confinement and release, as well as for directing the delivery of nanostructured cleaning fluids, such as micellar systems. Together, these features make semi-IPNs highly effective for applications demanding selectivity and controlled solvent action. Compared to conventional rigid gels or free solvent systems, they offer enhanced adaptability to complex substrates, reduced mechanical stress, and more precise control over cleaning performance [17,18,87].

Among these, PHEMA/PVP-based semi-IPNs represent the most extensively investigated platform, having been successfully applied to the controlled cleaning of a wide range of heritage materials, including stone, canvas, tempera paintings, and paper artefacts [69,70]. The incorporation of nanostructured fluids, typically in the form of micellar systems, further enhances cleaning selectivity by enabling the removal of oxidized or crosslinked varnishes while ensuring the stabilization and confinement of the micelles within the polymer network [17,18]. More recently, nanomagnetic PHEMA/PVP systems incorporating magnetite (Fe_3_O_4_) nanoparticles have emerged as advanced multifunctional systems, combining improved mechanical robustness and solvent retention with the possibility of remote manipulation, thus opening new perspectives toward stimuli-responsive cleaning strategies [88]. Magnetite nanoparticles are particularly attractive due to their biocompatibility, high magnetic responsiveness, and ease of synthesis and surface functionalization [89]. In this context, nanomagnetic hydrogels are typically obtained via γ-irradiation-assisted processes, enabling the simultaneous polymerization, crosslinking, and in situ immobilization of the nanoparticles within the polymer network.

PHEMA/poly(acrylic acid) semi-IPNs have been successfully applied to metallic substrates, where pH-responsive swelling and carboxylic functionalities enable selective copper ion chelation and efficient removal of corrosion products while preserving stable cuprite layers [90].

The Nanorestore Gel^®^ Peggy 5 consisting of a blend of PVA and PVP with enhanced hydrophilicity and tunable polarity expands compatibility with a wide spectrum of cleaning fluids, particularly those requiring higher solvent affinity or tailored interfacial interactions. The role of PVP is to expand solvent retention [91].

True interpenetrating polymer networks (IPNs) (Figure 2c), defined as systems composed of two or more independently crosslinked and physically interlaced polymer networks, represent an emerging yet still underexplored class of advanced soft materials for cultural heritage conservation. In contrast to semi-IPNs, where only one polymer is crosslinked, true IPNs—particularly double-network (DN) hydrogels—exhibit significantly enhanced mechanical robustness, resistance to deformation, and tunable transport properties arising from the synergistic interplay between rigid and ductile polymer networks [92,93]. Recent studies on stimuli-responsive IPN microgels, such as poly(N-isopropylacrylamide) and poly(acrylic acid) (PNIPAM/PAAc) systems, have demonstrated precise control over swelling/deswelling behavior and solvent diffusion, suggesting their potential as “smart” cleaning media capable of modulating solvent release in response to environmental triggers [94]. Furthermore, flexible PVA–borax-based gels, including poly(vinyl alcohol)–borax–agarose DN systems, have been systematically investigated in simplified model systems as advanced platforms for controlled cleaning applications [58,95]. Despite these promising features, the application of true IPNs in conservation practice remains limited, primarily due to challenges associated with their synthesis, scalability, and, critically, their reversibility, an essential requirement in conservation science. A summary of the key semi-IPN systems, treated artworks, performance, and advantages/disadvantages is provided in Table 4.

#### 2.2.4. Stimuli-Responsive Hydrogels

**Figure 8:**
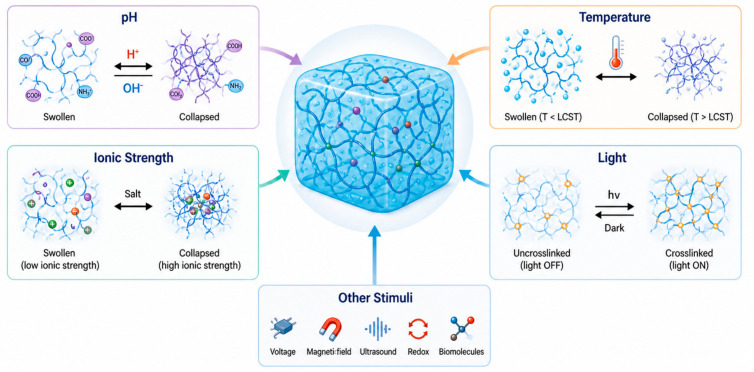
Schematic illustration of the main external stimuli regulating the reversible swelling and collapse of stimuli-responsive hydrogels. Changes in pH, ionic strength, temperature, and light induce reversible modifications in network structure through protonation/deprotonation, electrostatic screening, thermoresponsive transitions, or photo-induced crosslinking, respectively. Additional triggers, including electric voltage, magnetic fields, ultrasound, redox conditions, and biomolecules, can also modulate hydrogel properties and functionality.

Stimuli-responsive polymers, also referred to as “smart polymers,” are materials capable of undergoing reversible physicochemical changes in response to external stimuli, including temperature, pH, light, magnetic fields, and ionic strength [96,97]. These materials are particularly relevant for cultural heritage conservation because their adaptive behavior enables on-demand solvent release, selective adhesion, or mechanical actuation, reducing the risk of substrate damage [98] (Table 5 and Figure 8).

Among them, temperature-responsive polymers such as PNIPAM exhibit a well-defined lower critical solution temperature (LCST), allowing the polymer network to swell and retain aqueous cleaning fluids below LCST and expel solvent above LCST, providing a controllable cleaning cycle [99]. Similarly, pH-responsive polymers containing carboxylic acid or amine functionalities, such as poly(acrylic acid) or poly(2-(diethylamino)ethyl methacrylate), can modulate swelling and ion-binding behavior in response to local pH variations, which is highly advantageous for selective removal of corrosion products from metallic artefacts [90].

Magnetic or light-responsive semi-IPNs, integrating magnetic nanoparticles, typically magnetite, or photosensitive moieties, e.g., azobenzene moieties, offer remote-controlled gel positioning or activation, enhancing precision in complex cleaning procedures [88]. By combining stimuli-responsive behavior with the intrinsic tunability of semi-IPNs, these systems enable adaptive, non-invasive cleaning strategies.

**Figure 9:**
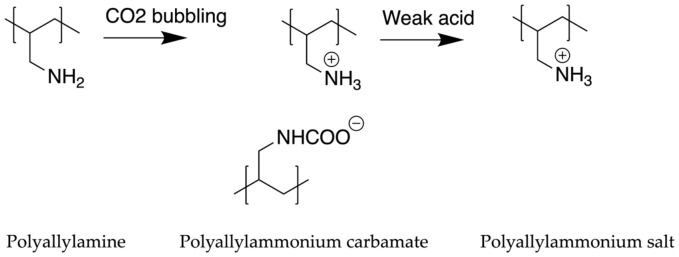
Synthesis of polyallylamine organogels [100].

A distinct class of responsive polymeric systems has emerged in which gel formation and disruption are governed by reversible chemical triggers rather than permanent or physical crosslinking. A representative example is based on polyallylamine, which acts as a latent gelling agent and undergoes CO_2_-induced transformation into polyallylammonium carbamate, enabling the formation of stable organogels in selected solvents under ambient conditions (Figure 9). This conversion induces a marked transition from liquid-like to solid-like rheological behavior [100]. Crucially, the process is fully reversible: mild acidification promotes CO_2_ release, rapidly restoring the system to a low-viscosity solution. Such chemically switchable gels provide a powerful platform for applications requiring dynamic control over viscosity and fluid confinement. In the context of art conservation, this behavior is particularly advantageous, as it allows the use of highly viscous, solvent-retaining gels during cleaning, followed by their rapid and complete removal through in situ viscosity reduction.

**Table 5:** Application of stimuli-responsive polymers in the cleaning of cultural heritage artworks.

Stimuli-Responsive Polymers	Artwork to Be Cleaned	Result	Advantages	Disadvantages/ Drawbacks	Reference
PNIPAM-based hydrogels (temperature- responsive)	Water-sensitive paintings Paper artefacts	Controlled swelling/ release of aqueous cleaning fluids	- On-demand solvent release - Reversible swelling - Minimal substrate penetration	- Limited to temperature-sensitive applications - LCST ^(a)^ tuning required	[99]
pH-Responsive poly(acrylic) SIPNs	- Bronze/copper alloys - Metal corrosion products	Removal of corrosion products	- Selective metal ion binding - Tunable swelling - It preserves stable patina	- pH must be carefully controlled - Limited to compatible substrates	[90]
Magnetic nanoparticle-loaded SIPNs	Experimental heritage models	Remote-controlled gel cleaning	- Non-contact control - Precise application	- Low technological readiness - It requires magnetic manipulation setup	[88]
Light-responsive polymers (azobenzene-functionalized networks)	Delicate painted surfaces	Photo-triggered swelling	- Remote activation - Precise application - Non-invasive	- Light penetration depth limitations - Early-stage technology	[101]
Multi-stimuli SIPNs (temperature + pH)	Mixed modern/contemporary artworks	- Dual-triggered solvent release - Cleaning selectivity	- Highly tunable - Adaptable to varying substrate sensitivities	- Complexity in design and synthesis - Cost-intensive	[102]

^(a)^ LCST: lower critical solution temperature.

#### 2.2.5. Polymeric Cleaning Systems in Cultural Heritage: A Critical Synthesis and Future Outlook

Functional polymers have become central to cultural heritage conservation. Hydrogels, organogels, and polymer-stabilized micro- and nanoemulsions provide tunable physicochemical properties for the selective treatment of diverse substrates and contaminants. Hydrogels enable controlled water-based cleaning of sensitive materials, organogels extend solvent action to hydrophobic layers, and nanoemulsions enhance solubilization while limiting substrate perturbation. Stimuli-responsive gels introduce on-demand control over diffusion, swelling, and release, further improving selectivity. No single polymer system is universally optimal; selection depends on substrate sensitivity, contaminant chemistry, and the required balance between efficiency, selectivity, and reversibility. These materials also support more sustainable practices by reducing chemical load, limiting hazardous waste, and improving operator safety. Future work will focus on bio-based polymers, multifunctional gels integrating cleaning, consolidation, and protection, hybrid composites with inorganic or nanostructured components, and standardized protocols to enable broader adoption.

### 2.3. Functional Polymers for the Consolidation of Artefacts

Polymeric consolidants are used in cultural heritage conservation to restore cohesion and mechanical strength in deteriorated porous materials [103,104]. Weathered stone, fragile wall paintings, porous ceramics, decayed wood, and powdery plaster commonly exhibit micro-fractures, delamination, and material loss because of aging processes, environmental exposure, or inappropriate past restoration treatments. Without consolidation, these weaknesses can progress, leading to irreversible damage.

Unlike adhesives, whose function is to create a bond between separate substrates, consolidants act within the material itself by reinforcing weakened microstructures.

**Figure 10:**
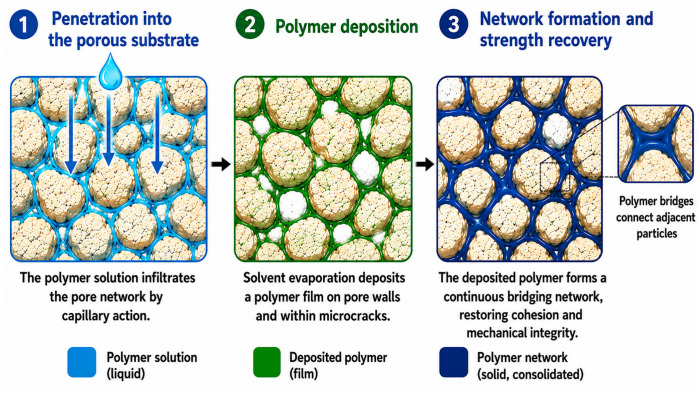
Schematic representation of the consolidation mechanism of polymeric consolidants in cultural heritage conservation, illustrating penetration into the porous substrate, polymer deposition, and formation of a continuous polymer network leading to the recovery of cohesion and mechanical strength.

The consolidation is governed by a sequence of physicochemical phenomena, progressing from polymer penetration and distribution to network formation and the recovery of cohesion within the degraded material (Figure 10). Following application, the consolidant penetrates the porous network through capillary forces (step 1). Once distributed within the substrate, the polymer is deposited on pore walls or between disconnected particles because of solvent evaporation, precipitation, curing, or polymerization processes (step 2). This generates new interactions within the degraded structure, including hydrogen bonding, van der Waals interactions, and, in some systems, covalent crosslinking. As the polymer network develops, bridges are established between adjacent particles and structural discontinuities are reduced, leading to an increase in cohesion and mechanical stability (step 3). The effectiveness of the treatment therefore depends on the ability of the consolidant to penetrate the substrate, interact with its internal surfaces, and form a stable reinforcing network without excessively altering porosity and transport properties.

Historically, consolidation treatments in cultural heritage conservation have relied on inorganic materials, such as limewater, ethyl silicate, and calcium hydroxide. Over time, synthetic polymeric consolidants have become increasingly important owing to their versatility and the possibility of tailoring their properties to specific conservation requirements.

Polymeric consolidants can be broadly classified into thermoplastic and thermosetting systems. Thermoplastic polymers generally offer greater re-treatability and ease of application, whereas thermosetting polymers provide superior mechanical reinforcement through the formation of crosslinked networks.

Among thermoplastic polymers, acrylic polymers have become the most extensively used consolidants because of their excellent optical transparency, chemical stability, compatibility with a wide range of substrates, and relatively good reversibility. Representative materials include Paraloid^®^ B-72 and related acrylic copolymers, which are among the most widely used consolidants for a broad range of cultural heritage materials [2,103,105]. Although their consolidating effect is generally confined to the superficial layers of stone, acrylic resins can still produce a significant improvement in the mechanical strength of the treated material [106]. Hybrid formulations combining alkoxysilanes with acrylic polymers have been widely investigated for the consolidation of stone [107].

In contrast, another thermoplastic polymer, poly(vinyl butyral) (PVB), has been employed in more specific consolidation treatments, particularly for degraded wooden artefacts and other porous organic substrates, owing to its good penetration ability and strengthening effect [108].

Thermosetting polymers, particularly epoxy resins, have been employed in applications requiring high mechanical strength and structural reinforcement, although their limited reversibility generally restricts their use [109,110].

Despite their effectiveness, conventional polymeric consolidants present some limitations. These include insufficient penetration into fine pore networks, potential embrittlement during long-term aging, aesthetic alterations such as yellowing or increased surface gloss, solvent-related health and environmental concerns, and, in some cases, limited re-treatability or reversibility. Moreover, the wide variability in the physicochemical properties of historic substrates requires consolidants with carefully tailored chemical composition, viscosity, molecular weight, and curing behavior.

The main polymeric systems investigated for cultural heritage conservation are summarized in Table 6.

### 2.4. Functional Polymers as Adhesives in Cultural Heritage Conservation

Polymeric adhesives are widely employed in cultural heritage conservation for the reassembly of fragmented objects, the reattachment of detached elements, and the stabilization of composite artefacts.

**Figure 11:**
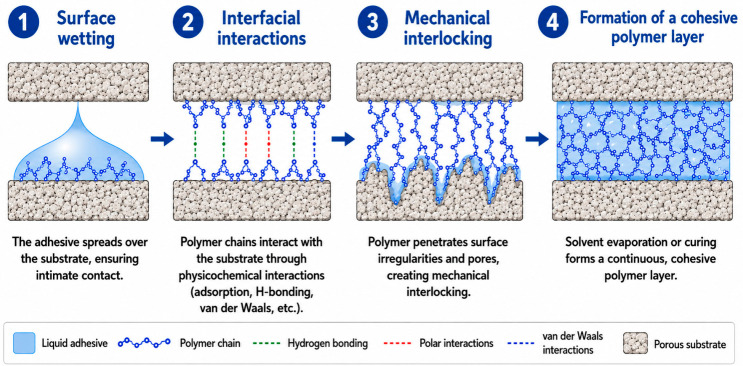
Schematic illustration of the main mechanisms governing the adhesion of polymeric materials in cultural heritage conservation, including wetting, interfacial interactions, chain interdiffusion, mechanical interlocking, and cohesive network formation.

Their effectiveness depends on both interfacial adhesion and the cohesive strength of the polymer, which together determine the strength and durability of the bonded system [111,112] (Figure 11). The adhesion process begins with the wetting of the substrate surface by the adhesive, which promotes intimate contact between the polymer and the substrate and maximizes the area available for molecular interactions (step 1). Depending on the chemical nature of the materials, adhesion may arise from hydrogen bonding, van der Waals forces, electrostatic interactions, acid–base interactions, or coordination phenomena involving surface functional groups (step 2) [113]. Polymer chains may further adsorb onto the substrate surface and, in porous materials, partially penetrate surface irregularities, generating mechanical interlocking (step 3). In polymeric substrates, chain interdiffusion across the interface may additionally contribute to bond formation. After solvent evaporation, cooling, or curing, the adhesive develops a continuous polymeric network that provides the cohesive strength required to withstand mechanical and environmental stresses (step 4). Consequently, the overall performance of a polymeric adhesive results from the combined contribution of interfacial adhesion and bulk cohesion.

A wide variety of polymeric adhesives has been employed in cultural heritage conservation, each offering distinct advantages depending on the substrate and conservation requirements (Table 7). Commonly used systems include acrylic polymers (e.g., Paraloid^®^ B72 [114,115,116,117]), PVAC [26], epoxy resins [115,118], and cellulose derivatives [27]. Their behavior depends on molecular architecture, glass transition temperature (T_g_), molecular weight distribution, and solvent–polymer interactions.

Acrylic copolymers, such as ethyl methacrylate/methyl acrylate systems, are widely used for their photochemical stability, transparency, and solubility in moderately polar solvents. PVAC formulations provide good adhesion and flexibility on porous substrates, whereas epoxy resins are preferred when high mechanical strength and structural consolidation are required, particularly for ceramics and stone.

However, long-term stability remains a major limitation. Degradation processes such as photo-oxidation, hydrolysis, chain scission, and plasticizer migration can lead to yellowing, embrittlement, and loss of adhesion, while irreversible penetration into porous substrates may hinder re-treatability.

To address these challenges, advanced polymeric adhesive systems with improved long-term stability, reversibility, and environmental compatibility are being actively developed. Current strategies include stimuli-responsive polymers, nanostructured and hybrid materials, and formulations based on green solvents or bio-derived polymers. Representative polymeric adhesive systems are summarized in Table 7.

### 2.5. Functional Polymers for the Surface Protection of Artefacts

Polymeric coatings are widely employed in cultural heritage conservation to protect artefacts from environmental degradation while minimizing alterations to their original appearance and properties [120]. Unlike consolidants and adhesives, which are designed to reinforce weakened structures or join detached elements, coatings act primarily at the material surface, creating a protective barrier that mitigates the interaction between the substrate and the surrounding environment.

The protective performance of a polymeric coating depends on its ability to modify surface properties of the substrate and limit the action of external degradation agents, including water, soluble salts, atmospheric pollutants, ultraviolet radiation, and biological contaminants [121]. Following application, the polymer wets the substrate and forms a thin protective layer through solvent evaporation, film formation, curing, or crosslinking processes, depending on the polymer chemistry and application method. This layer reduces the ingress of harmful species, thereby mitigating chemical, physical, and biological deterioration.

**Figure 12:**
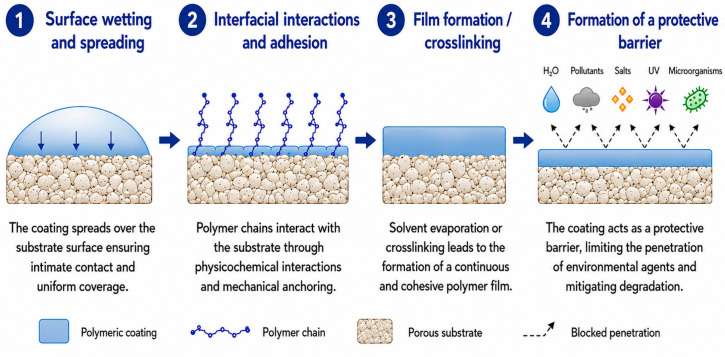
Schematic representation of the protective mechanism of polymeric coatings in cultural heritage conservation, illustrating surface wetting, interfacial adhesion, film formation, and the development of a protective barrier against environmental degradation agents.

The effectiveness of a polymeric protective treatment is governed by several physicochemical parameters, including surface energy, hydrophobicity, water vapor permeability, adhesion to the substrate, and resistance to environmental aging (Figure 12). An effective coating should form a thin, homogeneous barrier that limits the penetration of liquid water, soluble salts, and atmospheric pollutants while preserving the intrinsic pore structure and maintaining adequate water vapor permeability [122]. This requirement is particularly important for porous substrates, such as natural stone, where moisture transport is essential for the long-term durability of the material. Protective treatments should therefore avoid clogging surface pores or forming thick, continuous films that could hinder vapor diffusion, promote moisture accumulation, and accelerate deterioration. Instead, the coating should provide effective protection while allowing the substrate to remain breathable.

In accordance with the fundamental principles of cultural heritage conservation, polymeric protective systems should also exhibit good compatibility with the substrate and, whenever technically feasible, be removable or reversible [123], thereby enabling future re-treatment without causing irreversible alteration of the artefact.

The selection of an appropriate polymeric coating requires a multidisciplinary evaluation of its physicochemical, optical, and mechanical properties. Long-term stability is essential to prevent degradation phenomena such as yellowing, embrittlement, cracking, or loss of adhesion [124]. Optical neutrality is particularly important for decorated and polychrome surfaces, where even slight changes in color, gloss, or transparency may compromise the aesthetic perception of the artwork. Chemical and mechanical compatibility with the substrate must also be ensured to avoid adverse interactions, including solvent-induced damage, differential thermal expansion, or incompatible hygroscopic behavior [29].

This review examines polymeric coatings according to the type of artefact, addressing their application to stone, metals (e.g., bronze, copper alloys, and iron), polychrome and painted surfaces, ceramics and terracotta, and wood.

#### 2.5.1. Stone Surfaces: Case Studies

On stone surfaces, polymeric coatings are widely used to mitigate deterioration caused by weathering, atmospheric pollution, and moisture ingress, while preserving the original appearance of the substrate and maintaining adequate water vapor permeability. The main classes of polymeric systems employed for stone protection are summarized in Table 8.

Among them, conventional acrylic polymers, such as the Paraloid^®^ series, have long represented the benchmark protective coatings because of their ease of application, optical transparency and good protective performance [121,125]. More recently, fluorinated acrylic copolymers have attracted considerable attention owing to their superior hydrophobicity and enhanced resistance to photo-oxidative degradation [126,127,128,129,130]. The incorporation of fluorinated side chains lowers the surface energy of the coating, resulting in excellent water repellency while preserving the optical appearance of carbonate stones and marble.

In parallel, perfluoropolyethers (PFPEs) have been extensively investigated as protective coatings for stone because of their exceptional chemical inertness, low surface energy, and outstanding resistance to weathering and UV-induced degradation [131,132]. These properties enable the formation of durable hydrophobic barriers while maintaining adequate water vapor permeability, making PFPE-based systems particularly suitable for the protection of porous stone substrates. In addition, fluorinated oligoamide-based coatings have emerged as a new generation of protective materials [133,134]. By combining fluorinated segments with tailored polymer architectures, these systems provide excellent water repellency together with improved compatibility with stone substrates, preserving the pore network and minimizing aesthetic alterations while ensuring long-term protection against environmental degradation.

Recent research has further expanded this field through the development of hybrid organic–inorganic fluorinated coatings, which combine the durability of fluorinated polymers with the enhanced adhesion, mechanical stability, and substrate compatibility offered by inorganic components [135,136,137]. These multifunctional systems are designed to provide long-lasting protection while preserving moisture transport and the breathability of porous stone materials, which remain essential requirements for compatible conservation treatments.

**Table 8:** Polymers for the surface protection of stones in cultural heritage.

Polymer	Artwork to Be Coated	Results	Advantages	Disadvantages	References
Paraloid^®^ B72	Stone surfaces, e.g., limestone and marble	Water-repellence	- Good transparency - Ease of application - Removable with common solvents	- Relatively poor long-term durability outdoors due to UV light - Limited water resistance	[121,125]
Alkoxysilanes	- Sandstone - Limestone	- Surface hardness	Good chemical resistance	- It can cause surface cracking/shrinkage - Discoloration	[138]
Fluorinated acrylic polymers	- Limestone - Marble - Carbonate stones	- Hydrophobic coatings - Water repellence - Enhanced photostability	- Excellent water repellency - Improved weathering resistance - Good optical transparency	- Limited re-treatability after aging - Environmental concerns related to fluorinated monomers	[126,127,128]
Perfluoropolyethers	- Limestone - Marble - Porous stone substrates	- Durable hydrophobic protection - Water vapor permeability	- Excellent chemical and photo-oxidative stability - Minimal color alteration - High durability	- High cost - Difficult removal	[131,132]
Fluorinated oligoamides	- Historic stone - Carbonate stones	- Water repellency - Reduced capillary water uptake - Preservation of pore structure and vapor permeability	- Excellent compatibility with stone substrates - High photochemical stability - Durable protection - Lower environmental impact than conventional fluorinated systems	- Limited long-term field validation - Relatively recent technology	[133,134]

#### 2.5.2. Metals (Bronze, Copper Alloys, Iron): Case Studies

Polymer coatings on metallic artefacts are used to inhibit corrosion by isolating the surface from oxygen, moisture, and aggressive environmental agents [139]. Their protective action relies primarily on the formation of a barrier that limits the diffusion of water, dissolved salts, and atmospheric pollutants to the metal surface, thereby slowing the electrochemical processes responsible for corrosion. Among the most widely used systems, thermoplastic acrylic resins are appreciated for their transparency, ease of application, and relatively good reversibility, as they can generally be re-softened or removed using appropriate solvents. In contrast, thermosetting epoxy resins form highly crosslinked networks that provide excellent adhesion, chemical resistance, and barrier properties, resulting in effective long-term corrosion protection. However, the irreversible nature of the curing process significantly limits their removability and re-treatability, which may represent a drawback in conservation applications. The main systems are summarized in Table 9.

#### 2.5.3. Polychrome and Painted Surfaces: Case Studies

On polychrome and painted surfaces, polymer coatings are employed for both protection and consolidation, stabilizing fragile layers while preserving the aesthetic and historical integrity of the artwork. Depending on their formulation and application method, these materials can penetrate degraded paint films and porous substrates, restoring cohesion within the paint layer and improving adhesion between adjacent strata, while also providing protection against environmental factors. Acrylic polymers and PVAC systems [141] have been widely used owing to their good optical properties and ease of application, whereas waxes are often employed as protective coatings because of their water-repellent characteristics. Protein-based materials, such as gelatine and polysaccharides such as HPC and MC, remain important consolidants in specific conservation contexts due to their compatibility with traditional artistic materials. Since even minor visual alterations may affect the perception of an artwork, treatments must ensure high optical compatibility, long-term stability, and, whenever possible, reversibility. The main systems are summarized in Table 10.

#### 2.5.4. Ceramics and Terracotta: Case Studies

Polymer coatings for ceramics and terracotta are used to reduce porosity, limit water uptake, and mitigate surface degradation while maintaining visual and material compatibility. By penetrating the porous structure and/or forming a protective surface layer, these materials can improve cohesion, enhance mechanical stability, and reduce the ingress of moisture and soluble salts. Thermoplastic systems, such as acrylic polymers, are often preferred in conservation because of their ease of application and potential reversibility. The main systems are summarized in Table 11.

#### 2.5.5. Wooden Objects: Case Studies

Polymer coatings on wooden artefacts protect against moisture fluctuations, biological attack, and mechanical damage, thereby enhancing long-term material stability [151]. These materials act by reducing the exchange of moisture between the wood and the surrounding environment, limiting dimensional changes that may lead to cracking, warping, or surface deterioration. In addition to providing a protective barrier, some polymeric systems can improve the cohesion and mechanical integrity of degraded wood. Given the hygroscopic nature of wood, particular attention must be paid to maintaining compatibility with the substrate and avoiding excessive restriction of moisture transport. The main polymers used for wood protection are acrylic polymers and epoxy resins, whose main characteristics are described in Table 12 [152,153,154,155,156,157]. In addition, a recent review of bio-based acrylic coatings for cultural heritage conservation has highlighted their superior performance compared with conventional fossil-based materials [154].

#### 2.5.6. Polymer Coatings in Cultural Heritage: A Comparative Synthesis and Critical Assessment

Polymeric coatings play a key role in the protection and stabilization of cultural heritage artefacts by providing resistance to chemical degradation, environmental exposure, and mechanical stress. Over recent decades, a wide range of materials has been developed, including acrylic and vinyl polymers, biopolymers, and, more recently, hybrid organic–inorganic networks and nanostructured coatings. Their performance depends on physicochemical properties such as optical transparency, penetration capability, reversibility, hydrophobicity, UV resistance, and long-term stability, which must be tailored to the characteristics of specific substrates, e.g. metals, stone, ceramics, polychrome surfaces, and wood. Importantly, in conservation practice, long-term stability often needs to be balanced against reversibility requirements.

The available coating systems differ significantly in terms of durability, compatibility, and transport properties, reflecting differences in polymer architecture and interfacial behavior. Acrylic and vinyl polymers remain among the most widely used materials due to their transparency, ease of application, and relative reversibility. However, prolonged exposure to UV radiation, temperature fluctuations, and humidity can induce photo-oxidative degradation, chain scission, embrittlement, and changes in solubility, progressively compromising both protective performance and visual compatibility. Fluoroacrylic formulations provide improved hydrophobicity and weathering resistance owing to the high stability of fluorinated segments, but their environmental persistence and the increasing concerns associated with fluorinated compounds raise questions regarding their long-term sustainability.

These limitations have stimulated the development of alternative systems designed to better balance durability and compatibility. Silane- and siloxane-based treatments enhance hydrophobicity through the formation of inorganic networks within porous substrates, although environmental exposure may progressively alter network integrity and reduce protective effectiveness. Similarly, nanocellulose-based and chemically modified biopolymers have shown considerable promise for the consolidation of wooden artefacts by combining mechanical reinforcement with lower chemical impact and improved reversibility. Nevertheless, their degradation pathways and long-term behavior under fluctuating environmental conditions remain insufficiently understood.

Overall, the comparison of different coating families reveals a recurring trade-off between environmental stability and re-treatability. Systems characterized by strong intermolecular interactions, high crosslink density, or highly hydrophobic structures generally exhibit superior durability but often reduced reversibility. Conversely, materials designed to facilitate removal and re-treatment may be more susceptible to aging and environmental degradation. This balance represents one of the major challenges in the design of conservation polymers and explains why no single coating system can be considered universally suitable.

From a polymer science perspective, the different behaviors observed among coating systems can largely be attributed to variations in molecular weight, crosslink density, interfacial adhesion, and vapor transport properties. Acrylic and vinyl polymers generally favor reversibility and optical compatibility but are more vulnerable to photo-oxidative degradation, whereas silane- and siloxane-based systems provide enhanced moisture resistance for porous substrates. Bio-based materials offer advantages in terms of sustainability and substrate compatibility but still require further validation of their long-term performance. Material selection must therefore be guided by the relationship between polymer structure, degradation mechanisms, and substrate-specific requirements.

Sustainability is becoming an increasingly important consideration in the development of conservation materials. The growing interest in renewable and biodegradable polymers reflects the need to reduce environmental impact while maintaining adequate performance. At the same time, the environmental persistence of fluorinated materials highlights the importance of evaluating coating systems not only in terms of durability but also with respect to their life-cycle impact and long-term environmental compatibility.

Despite significant advances, several challenges continue to limit the effectiveness of polymeric coatings. The degradation of conservation polymers is governed by multiple and often interconnected processes, including photo-oxidation, hydrolysis, network restructuring, and moisture-induced stress development. Their relative importance depends on both polymer architecture and substrate characteristics. In porous materials, differences in thermal expansion and moisture transport between the coating and the substrate can generate internal stresses, leading to cracking, delamination, and loss of adhesion. Consequently, coating failure is rarely controlled by a single degradation mechanism but rather results from the interplay between polymer chemistry, interfacial interactions, environmental exposure, and substrate response.

This complexity highlights the limitations of conventional accelerated aging studies, which often assess degradation factors independently despite the simultaneous environmental stressors experienced by cultural heritage materials. Future research should prioritize realistic multifactor aging protocols and establish quantitative relationships between polymer structure, degradation mechanisms, and long-term performance. Advancing this structure–property–performance framework will be essential for the rational design of next-generation conservation coatings.

## 3. Polymers as Constituent Materials in Artefacts

Polymers constitute a major class of materials in cultural heritage artefacts, including both natural and synthetic types. Natural polymers, mainly polysaccharides such as cellulose, are key components of paper, historical textiles and wood, while synthetic polymers have become increasingly widespread in modern and contemporary art. These materials play a fundamental role in determining the structural, surface, and aesthetic properties of artefacts. However, their organic nature makes them especially prone to chemical, physical, and environmental degradation, creating significant challenges for their preservation and conservation.

### 3.1. Natural Polymers

#### 3.1.1. Cellulose-Based Artefacts

Cellulosic materials, including paper and textiles, represent a major category of cultural heritage artefacts. Their preservation is challenging due to the intrinsic chemical and physical instability of cellulose, which is susceptible to hydrolytic, oxidative, and biological degradation [158]. The deterioration of these materials is governed by multiple, often interrelated, factors.

Acid-catalyzed hydrolysis, promoted by pollutants (e.g., SO_2_, NOx) [159], (potassium aluminum sulfate) sizing, residual inks, or degradation products, leads to cleavage of glycosidic bonds, resulting in a reduced degree of polymerization, loss of mechanical strength, and embrittlement, while oxidative processes driven by UV light exposure, oxygen, and trace metals induce chain scission and the formation of carbonyl and carboxyl groups, further accelerating degradation and contributing to yellowing [160]. In addition, fluctuations in relative humidity and temperature cause dimensional changes, generating mechanical stress, warping, and microcracking, particularly in layered or composite structures such as painted canvases and manuscripts. Biological activity also plays a significant role, as fungi and bacteria enzymatically degrade cellulose, especially under high humidity conditions, leading to surface weakening, staining, and fiber loss [161].

Effective conservation of cellulosic artefacts requires stabilizing the polymeric structure while preserving their aesthetic and functional properties. Treatments must penetrate fragile, porous substrates without inducing structural collapse, avoid introducing harmful residues, and maintain optical, tactile, and mechanical integrity [162]. Both natural and synthetic polymers have been widely employed for the consolidation, protection, and stabilization of cellulosic materials in cultural heritage, offering distinct advantages in terms of compatibility, durability, and reversibility, while also presenting specific limitations related to aging and environmental sensitivity (Table 13).

#### 3.1.2. Paper: Case Studies

Paper, the primary support for manuscripts, drawings, and archival materials, is mainly composed of cellulose fibers, responsible for tensile strength, flexibility, and dimensional stability [2]. During manufacture, additives such as starch, gelatine, and alum are introduced to improve surface properties, ink receptivity, and handling [167]. While these components enhance functionality, they may also affect long-term chemical stability, particularly under environmental stress. As already discussed in Section 3.1.1, cellulose decomposition proceeds through hydrolytic and oxidative pathways, accelerated by acidic conditions, fluctuations in relative humidity, light exposure, and atmospheric pollutants, ultimately causing yellowing, embrittlement, and fiber weakening [168].

Writing and drawing media represent a primary driver of paper degradation, with iron gall inks standing out as particularly deleterious agents. Ubiquitous from the Middle Ages to the nineteenth century, these inks, formulated from iron(II) sulfate, tannic and gallic acids, and a polysaccharide binder (typically gum Arabic), impart not only intense coloration but also long-term chemical instability. Their incorporation into the paper matrix introduces both acidity and redox-active iron species, which synergistically catalyze cellulose depolymerization via acid hydrolysis and iron-mediated oxidative pathways. The cumulative effects manifest as embrittlement, cracking, ink corrosion (burn-through), and ultimately the irreversible loss of textual and graphical information [169,170]. Conservation strategies therefore target the concurrent stabilization of both the ink and the cellulose substrate. Current protocols typically integrate phytate-based treatments, which sequester catalytically active iron species, with calcium bicarbonate deacidification to neutralize acidity and establish an alkaline reserve. When judiciously applied, these interventions markedly retard degradation while preserving legibility and visual integrity. However, their efficacy critically depends on stringent process control, as improper application may induce ink solubilization, redistribution, or mechanical stress within the weakened paper matrix [169,170].

Table 14 summarizes representative case studies on the degradation and conservation of cellulosic artefacts, with particular emphasis on the relationship between degradation mechanisms, treatment approaches, and the use of polymer-based materials.

#### 3.1.3. Wood-Based Artefacts: Case Studies

Wood is a hierarchically organized natural composite comprising cellulose, hemicellulose, and lignin, whose multiscale polymer architecture underpins its mechanical strength and resilience [179]. Semi-crystalline cellulose microfibrils embedded within an amorphous hemicellulose–lignin matrix govern structure–property relationships while simultaneously defining the principal pathways of degradation in cultural heritage artefacts, including sculptures, panel paintings, architectural elements, and furniture. Environmental deterioration is largely driven by the intrinsic hygroscopicity of polysaccharides: repeated sorption–desorption cycles induce anisotropic swelling and shrinkage, generating internal stresses between crystalline and amorphous domains. These processes ultimately promote microcracking, interfacial decohesion, and progressive mechanical fatigue [155,180].

Chemical decomposition proceeds through coupled hydrolytic and oxidative pathways that progressively undermine the wood polymer network. Acid- or base-catalyzed hydrolysis cleaves glycosidic linkages in hemicelluloses and cellulose, lowering the degree of polymerization and disrupting load transfer within the structural framework [158]. In parallel, metal-catalyzed oxidative processes induce chain scission and chemical transformation across both polysaccharides and lignin, driving embrittlement and the formation of chromophoric species. At exposed surfaces, UV-driven photodegradation further accelerates lignin depolymerization, amplifying structural weakening and discoloration [181].

Biological deterioration of wood is driven by enzymatic depolymerization processes carried out by fungi, bacteria, and insects [182]. Brown-rot fungi selectively degrade polysaccharides, whereas white-rot fungi can mineralize lignin [183]. Under conditions of high moisture, soft-rot fungi and bacterial activity induce the formation of microcavities within the cell wall, increasing material permeability; concurrently, the activity of xylophagous insects creates preferential pathways that facilitate further microbial colonization and moisture ingress.

Understanding the interplay among these interconnected degradation mechanisms is essential for designing effective conservation strategies aimed at preserving both the polymeric structure of wood and the cultural value of the objects themselves [155]. While the deterioration mechanisms described above are common to most wooden artefacts, waterlogged archaeological wood represents a particularly challenging case due to the extensive loss of structural polysaccharides and the consequent collapse of the cell wall upon drying.

Polyethylene glycol (PEG)-based treatments remain the most widely adopted approach for the conservation of waterlogged archaeological wood because of their ability to replace water within the degraded cell wall and improve dimensional stability during drying [184]. Nevertheless, concerns related to long-term stability, re-treatability, and environmental sustainability have stimulated the search for alternative consolidation systems.

Increasing attention has been devoted to bio-based materials as sustainable and compatible consolidants. Cellulose derivatives, including hydroxyethyl cellulose and chitosan, have been investigated because of their compatibility with the wood cell wall, biodegradability, and ability to improve dimensional stability while minimizing adverse effects on the original material [185]. Other promising bio-based consolidants include low-molar-mass chitosan and medium-molar-mass alginate, which have demonstrated good penetration into degraded wood, effective stabilization of weakened cell walls, and favorable compatibility with archaeological substrates [186].

Alongside these materials, nanostructured systems have emerged as promising alternatives for the conservation of waterlogged wood. Cellulose nanocrystals (CNCs) and bacterial nanocellulose have demonstrated excellent potential for reinforcing degraded cell walls owing to their high mechanical strength, nanoscale dimensions, and compatibility with the wood ultrastructure [187]. In parallel, lignin-based systems, including lignin nanoparticles and chemically modified lignin formulations, have attracted increasing attention because they exploit the intrinsic chemical affinity of lignin with the residual lignified cell wall, thereby improving consolidation efficiency while maintaining excellent compatibility with the original substrate. Collectively, these bio-based, nanostructured, and lignin-derived materials considerably broaden the range of sustainable conservation strategies available for waterlogged archaeological wood and represent promising alternatives or complementary treatments to conventional PEG-based systems [188,189,190].

Table 15 presents a critical analysis of conservation treatments applied to waterlogged ships, among the most extensively studied wooden artefacts owing to the urgency of the required interventions.

#### 3.1.4. Wooden Musical Instruments: Case Studies

Wood is a fundamental material in the construction and performance of musical instruments, where its polymeric architecture governs tonal quality, resonance, and mechanical stability [179,202]. The acoustic performance of violins, cellos, guitars, pianos, harpsichords, and pipe organs depends on density, fiber orientation, hemicellulose content, and resin composition. Wood is sensitive to environmental fluctuations, UV-induced oxidation, biological degradation, and mechanical stress, (e.g., string tension, bowing, or hammer impact), which generate microfractures and progressive fatigue over time.

Natural aging, while typically detrimental to structural stability, can enhance the acoustic performance of wood. Progressive hemicellulose depletion, coupled with increased cellulose crystallinity, alters density and stiffness, leading to improved vibrational response, sustain, and harmonic richness [202,203]. These effects are primarily driven by hemicellulose hydrolysis and lignin oxidation, while crystalline cellulose remains largely unaffected [204]. Consistently, thermally and UV-aged spruce and maple exhibit increased sound velocity and Young’s modulus, reflecting enhanced stiffness and acoustic efficiency in tone wood [205]. Evidence from aged Cremonese maple used in historical violins confirms this trend, linking hemicellulose degradation and lignin oxidation, alongside preserved cellulose crystallinity, to the distinctive acoustic qualities of these instruments [206].

Together, these observations define an “acoustic maturation paradox,” wherein controlled polymer degradation improves, rather than compromises, acoustic performance. This duality underscores the need for conservation strategies that stabilize the polymer network while preserving its time-dependent evolution [204,206]. Accordingly, optimal approaches combine controlled environmental conditions (relative humidity ~40–50%, temperature ~18–22 °C), minimal surface intervention, compatible reinforcement materials (e.g., cellulose ethers or resin-based fillers), and reversible adhesives such as hot hide glue, thereby maintaining both structural integrity and acoustic function [202,203].

Table 16 provides a list of case studies of wooden musical instruments highlighting wood type, degradation, and acoustic outcomes.

### 3.2. Synthetic Polymers in Cultural Heritage

#### 3.2.1. Historical Development of Plastics in Art and Design

The history of plastics in art and design originates in the late nineteenth century, when early semi-synthetic materials such as Parkesine^®^ [211] and Celluloid^®^ [212] were developed as substitutes for scarce natural resources including ivory and tortoiseshell. Their moldability, color versatility, and suitability for mass production opened new possibilities for decorative arts, photography, and early consumer goods, marking the beginning of a profound material transformation.

A decisive milestone was reached in 1907 with the invention of Bakelite [213], the first fully synthetic plastic. Its heat resistance, structural stability, and capacity for vibrant coloration revolutionized manufacturing and design. Bakelite objects, ranging from telephones and radios to jewelry and kitchenware, embodied the emerging visual language of modernity, where industrial production and aesthetic innovation became increasingly intertwined.

During the mid-twentieth century (1930–1960), the diffusion of polymers such as acrylics, PVC, and polyethylene (PE) consolidated plastics as emblematic materials of post-war progress. Designers embraced their lightness, flexibility, and affordability, integrating them into domestic environments that projected efficiency, modernity, and accessibility. Plastics became synonymous with a democratized vision of design.

From the 1960s to the 1980s, plastics entered contemporary artistic practice with unprecedented intensity. Within the context of Pop Art and experimental sculpture, materials such as resins, plexiglass, and synthetic fibers were employed to produce transparent, glossy, and vividly colored works. In this phase, plastics functioned not only as materials but also as cultural signifiers, reflecting consumerism, artificiality, and the shifting perception of objects in industrial society.

Between 1980 and 2000, increasing awareness of environmental issues transformed the cultural meaning of plastics. Once celebrated as symbols of progress, they became associated with pollution and excess. Artists began to incorporate discarded plastics, industrial waste, and found materials into assemblages and installations, critically engaging with themes of overproduction, environmental degradation, and material afterlife.

In the twenty-first century, artistic and design practices are characterized by a renewed engagement with sustainability. This includes the use of recycled and up-cycled plastics, the development of bio-based and biodegradable polymers, and the adoption of digital fabrication technologies such as 3D printing. At the same time, growing attention has been devoted to the conservation of plastic artefacts, whose inherent instability poses significant challenges to traditional museum practices.

#### 3.2.2. Plastics in Art and Design: Materials, Properties, and Conservation

In parallel with their historical development, synthetic polymers have progressively assumed a central role as primary materials in modern and contemporary artistic practice. Beyond their traditional use as adhesives and protective coatings, plastics and resins have enabled a radical rethinking of artistic materials, processes, and aesthetics. Since the mid-twentieth century, artists have adopted polymers not only for their technical advantages but also for their conceptual implications, embracing impermanence, seriality, transparency, and industrial production as integral components of artistic expression [4]. Their relevance in artistic practice is rooted in structure–property relationships. Parameters such as molecular weight distribution, degree of crosslinking, glass transition temperature (T_g_), and additive content (e.g., plasticizers) govern processability, optical transparency, viscoelastic response, and long-term stability. Commonly employed polymers include PMMA, PU, epoxy resins, and PVC, each characterized by specific chemical and physical decomposition pathways that directly influence both artistic intent and conservation strategies [214].

PMMA is an amorphous glassy polymer (T_g_ ~ 105 °C) whose optical transparency derives from the absence of crystalline domains and minimal light scattering. Under UV exposure, PMMA undergoes photo-oxidative decomposition via main chain scission, leading to molecular weight reduction, decreased T_g_, and the formation of chromophores responsible for yellowing. These changes are accompanied by increased chain mobility and enhanced susceptibility to surface abrasion and microcracking [215]. Artists, such as Donald Judd, employed PMMA, often marketed as Lucite^®^ [216], to create precise, modular forms whose perceptual impact is defined by reflections, refractions, and the interaction with natural and artificial light [217].

Polyurethanes encompass a wide range of materials, from elastomeric systems to highly crosslinked networks, depending on isocyanate–polyol chemistry. Their properties arise from phase-separated morphologies in which soft and hard segments control viscoelastic behavior. However, PUs are particularly prone to degradation, including hydrolysis of ester linkages, oxidative reactions, and additive migration, resulting in chain scission, loss of mechanical integrity, and progressive embrittlement [214]. The sculptures of Eva Hesse exemplify the expressive potential of PU, especially its capacity to evoke organic, process-oriented, and inherently unstable forms [218,219].

Epoxy resins are highly crosslinked thermosetting polymers formed via epoxide ring-opening reactions, characterized by high mechanical strength and limited chain mobility. While these features confer dimensional stability, they also promote internal stress accumulation. Under UV exposure and thermal cycling, epoxy networks undergo photo-oxidation and structural rearrangements, leading to yellowing, microcracking, and stress-induced damage [220].

Artists such as Ann Veronica Janssens have used epoxy resins to encapsulate objects or pigments within transparent or translucent volumes, creating immersive environments that explore perception, color, and light [221,222].

PVC is a thermoplastic polymer whose properties are strongly dependent on formulation, particularly plasticizer content. In flexible systems, plasticizers reduce intermolecular interactions and lower T_g_, enabling high flexibility. Over time, however, plasticizer migration and evaporation lead to increased T_g_, loss of flexibility, and surface tackiness. Concurrently, dehydrochlorination reactions under thermal or UV stress generate conjugated polyene sequences, resulting in discoloration and accelerated degradation [223]. Contemporary artists, such as Jeff Koons, have exploited PVC for its glossy surfaces and industrial aesthetic, engaging with themes of consumer culture and mass production [224,225].

While synthetic polymers have profoundly expanded the material possibilities of contemporary art, their use also introduces complex and often irreversible conservation challenges. Phenomena such as yellowing, microcracking, embrittlement, loss of flexibility, and chemical migration are not merely technical issues, but factors that directly affect the meaning, perception, and longevity of artworks.

As a result, conservation of polymer-based artworks requires a materials-driven approach grounded in polymer chemistry and physics.

At the same time, this intrinsic instability is not merely a technical limitation but part of the cultural significance of polymeric materials. The tension between durability and decay is embedded in their molecular nature, where controlled architectures coexist with evolving degradation pathways. As Andy Warhol famously remarked, *“Everybody’s plastic, but I love plastic. I want to be plastic.”* This observation captures the dual role of polymers as engineered materials and cultural constructs, situated at the intersection of industrial production, material instability, and evolving artistic meaning.

Table 17 presents selected examples of artists and designers who employed polymeric materials in their artefacts.

#### 3.2.3. Polymer-Based Cultural Heritage: A Critical Analysis of the Current State of Art in the Conservation of Plastics in Art and Future Perspectives

Recent advances in polymer science have broadened both the diversity and complexity of materials used in contemporary artworks, including multi-component systems and polymers with tailored functionalities. For conservation, this underscores a key point: polymeric materials cannot be adequately understood through nominal composition alone. Their behavior reflects the combined effects of molecular structure, additives, processing history, and morphology, all of which govern degradation pathways and rates. Despite progress in analytical methods and accelerated aging protocols, predicting long-term behavior under real conditions remains a major challenge. Accelerated aging often fails to capture the variability observed in historical objects, particularly when formulations are unknown or when processing-induced heterogeneities are significant. Existing models are typically system-specific and rarely transferable across the wide range of polymer-based artefacts. As a result, conservation practice still relies heavily on detailed material characterization and empirical evidence, with limited predictive capability.

A central priority is therefore the development of robust structure–property–degradation relationships that enable more reliable predictions over time. This will require not only improved analytical tools, but also systematically generated and comparable datasets linking formulation, processing, and environmental exposure to measurable degradation outcomes, an area that remains underdeveloped.

At the same time, the material landscape continues to evolve. Plastics are not a fixed class of materials, but an expanding one, increasingly adopted by artists for their versatility. Conservation must therefore address not only legacy materials, but also newly designed polymer systems whose long-term behavior is often unknown.

In this context, effective conservation demands a polymer science-based approach: case-specific analysis, strict control of environmental conditions, and cautious interpretation of aging data. The role of the polymer chemist is not only to characterize degradation, but also to define the limits of prediction and provide a sound basis for conservation decisions.

## 4. Conclusions and Future Perspectives

Polymers have become foundational materials in cultural heritage conservation, shaping both practical treatment methodologies and broader conservation strategies. Established systems, particularly acrylic polymers, epoxy resins, polyvinyl acetate, and cellulose derivatives, remain indispensable for a wide range of interventions, including consolidation of fragile substrates, adhesive treatments, protective coatings, surface stabilization, and cleaning operations. Their optical clarity, chemical stability, and mechanical compatibility have enabled their successful application across diverse materials such as stone, wood, paper, textiles, wall paintings, and contemporary artworks. Their widespread use reflects decades of proven performance, but also a historical reliance on materials whose long-term evolution is not always fully predictable.

The analysis presented in this review highlights that the effectiveness of polymer-based treatments depends not only on the intrinsic properties of the materials employed but also on their interaction with specific substrates, environmental conditions, and aging processes. No single polymer can be considered universally suitable for all conservation needs, emphasizing the importance of case-specific material selection supported by scientific characterization and performance evaluation.

Accumulating evidence demonstrates that polymer treatments are neither inert nor permanent. Degradation processes, including photo-oxidation, hydrolysis, additive migration, chain scission, and crosslinking, can progressively alter their physical and chemical properties, leading to embrittlement, discoloration, loss of adhesion, reduced reversibility, and, in some cases, secondary damage to the original substrate. These phenomena underline one of the central challenges in conservation practice: balancing immediate treatment effectiveness with long-term stability, compatibility, and re-treatability.

Recent advances in polymer science have significantly expanded the range of available conservation tools. Nanostructured polymers, hydrogels, organogels, and polymer-stabilized emulsions have transformed cleaning methodologies by enabling controlled solvent release, selective removal of unwanted materials, and reduced interaction with sensitive surfaces. Similarly, innovative consolidants and protective formulations provide improved penetration, adhesion, and durability while minimizing undesirable side effects. Stimuli-responsive polymers and tailored multifunctional systems further enhance treatment selectivity and adaptability, supporting more precise and substrate-specific interventions.

At the same time, sustainability has emerged as an increasingly important criterion in material selection. Bio-based, biodegradable, and recyclable polymers offer promising alternatives to conventional petrochemical products, allowing conservation practice to align with broader environmental objectives without compromising effectiveness. Although further studies on their long-term behavior are still required, these materials represent a significant step toward more sustainable conservation strategies.

Polymers also constitute cultural heritage in their own right, particularly within modern and contemporary art, where inherent material instability challenges traditional concepts of permanence, authenticity, and conservation intervention. Addressing these issues requires an interdisciplinary approach that combines polymer chemistry, materials science, conservation practice, and critical reflection on the cultural significance of material change.

Looking ahead, future progress will depend on the integration of chemical knowledge, technological innovation, and ethical awareness. While established polymer systems will continue to play a central role in conservation treatments, emerging materials, particularly bio-based, nanostructured, and stimuli-responsive polymers, offer new opportunities for safer, more selective, and environmentally responsible interventions. Combined with advanced analytical techniques, predictive aging studies, and tailored formulations, these developments will enable increasingly informed conservation strategies capable of responding to the specific needs of both traditional and contemporary cultural heritage materials.

Ultimately, polymers occupy a unique intersection of science, ethics, and cultural interpretation. Their versatility represents both an opportunity and a responsibility. Future developments must continue to balance performance, reversibility, sustainability, and long-term compatibility while acknowledging the evolving cultural meanings of the materials being preserved. In this context, polymers are not merely conservation products but active mediators in the preservation, transformation, and transmission of cultural heritage through time.

## Figures and Tables

**Table 1:** Polymers for the pre-consolidation artworks in cultural heritage.

Polymer	Artwork to Be Pre-Consolidated	Application Method	Advantages	Limitations/Drawbacks	Reference
PVAC-based solutions or gels	- Friable stone - Painted surfaces	It can be applied as a thin gel sheet for controlled pre-consolidation	- High reversibility - Minimal solvent exposure	-	[26]
Hydroxypropyl methylcellulose (HPMC) or methylcellulose (MC)	Water-sensitive surfaces	Formulate into thin, low-viscosity gels for selective application, it can gently bind loose particles	- Non-toxic - Easy removal - Tunable viscosity - Suitable for delicate mural or paper surfaces	- Lower mechanical strength than synthetic resins - Possible microbial growth if residues remain	[27]
Low molecular weight acrylics, e.g., Paraloid^®^ B72 ^(a)^ in dilute solutions	Friable pigments before cleaning	Applied in low-concentration solutions (1–3% *w*/*v* in ethanol or acetone) to penetrate fine cracks without altering optical properties	- Good reversibility - Low yellowing - Minimal mechanical impact	- Solvent exposure - Risk of uneven distribution if not carefully applied	[28]

^(a)^ Paraloid^®^ B-72 is a copolymer of ethyl methacrylate (EMA) and methyl acrylate (MA), with an approximate EMA:MA monomer ratio of 70:30. The representative repeating unit of Paraloid^®^ B-72 is shown below. 
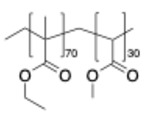

**Table 3:** Application of organogels in the cleaning of cultural heritage artworks.

Organogels	Artwork to Be Cleaned	Result	Advantages	Drawbacks	References
Nanorestore^®^ Gel	- Varnished paintings - Oil paintings - Contemporary art	Removal of: - varnish -grime	- Excellent control of solvent diffusion - Minimal surface stress - Very little residue	- More expensive - It requires correct matching of gel strength to substrate	[12]
Polysiloxane-based gels	- Wall paintings - Polychrome sculptures	Removal of: - waxes - aged coatings - hydrophobic grime	- Excellent solvent retention - Non-polar environment - Easy peel-off	- Very hydrophobic unsuitable for polar contaminants - Limited mechanical flexibility	[78,79,80]
Cyclododecane organogels	- Stone - Frescoes - Archaeological objects	-	- Reversible by sublimation - Useful barrier to protect sensitive surfaces	Slow sublimation in cold environments	[81,82]
Solvent-emulsion organogels	- Oil paintings - Water-sensitive surfaces	Highly controlled release of mixed polar–non-polar solvents	- High cleaning selectivity - Adjustable viscosity	- Delicate preparation - Stability varies with formulation	[83]
Ethylene–vinyl acetate (EVA)/ethyl-lactate organogels	- Modern paintings - Sensitive acrylic surfaces	Removal of: -plasticizer - grime	- Mild action - Green solvent combinations	It can soften acrylic paints if left too long	[84]
Hydrophobic PU organogels	- Metal artefacts - Soil deposits on mixed materials	Removal of: - oily grime - tar residues	Strong mechanical integrity	- Risk of over-cleaning - It can be too aggressive	[70]
Organogelated surfactant systems	Painted surfaces	Removal of: -oily grime - aged natural resins	- High chemical efficiency - It can be tuned with pH	- Requires careful managing - It may leave surfactant traces	[78]
Gellan-gum organogels with organic solvents	- Paper - Parchment	Removal of hydrophobic stains	- High dimensional stability on paper - Low water penetration	Limited solvent loading	[85]

**Table 4:** Application of semi-interpenetrating and interpenetrating polymer networks in the cleaning of cultural heritage artworks.

SIPNs	Artwork to Be Cleaned	Result	Advantages	Drawbacks	Reference
pHEMA/PVP	- Water-sensitive paintings - Paper artefacts	Removal of: - dirt - coating	- High solvent confinement - Mechanical stability	Limited for hydrophobic varnishes	[17]
pHEMA/PVP	Painted artistic surfaces	Cleaning efficiency	- Tunable porosity - Reproducibility	Complex synthesis	[87]
Nanomagnetic pHEMA/PVP	Experimental heritage models	Cleaning	Multifunctionality	Early-stage	[88]
pHEMA/PVP + nanostructured fluids	Classical oil paintings	Removal of varnish	- High selectivity - Reduced solvent toxicity	Complex formulation	[18]
pHEMA/	Bronze and copper alloys	Removal of corrosion products	- Chelating activity - pH tunable	Limited to metals	[90]
poly(acrylic acid)					
PVA + nanofluids	Picasso’s “L’Atelier”	Removal of varnish	Preserves original stratigraphy	Requires careful tuning	[54]

**Table 6:** Polymers for the consolidation of artworks in cultural heritage.

Polymer	Artwork to Be Consolidated	Application Method	Advantages	Limitations/Drawbacks	References
Acrylic polymers, e.g., Paraloid^®^ B72, B48N ^(a)^, Acryloid^®^ F-10 ^(b)^	- Friable frescoes - Easel paintings - Stone sculptures	Applied as low- to medium-viscosity solutions in ethanol or acetone	- Excellent adhesion to diverse substrates - Reversible with organic solvents - Chemically stable - Minimal color change	- Solvent use - Potential penetration issues in highly porous materials	[2,103,105,107]
Poly(vinyl butyral) + ZnO nanocomposite	Wooden artefacts		- Excellent film-forming properties - Elastic consolidation - Reduced risk of cracking in fluctuating environmental conditions	- Low solubility in green solvents - Careful formulation needed to ensure reversibility	[108]
Epoxy and PU-based polymers	- Decayed wooden beams - Stone sculptures with fractures		- High mechanical strength - Adhesion to substrates - Resistance to moisture and temperature changes	- Generally irreversible - Less compatible with delicate artworks - Potential long-term chemical aging	[109,110]

^(a)^ Paraloid^®^ B48N is a copolymer of methyl methacrylate (MMA) e butyl acrylate (BA) with 80:20 molar ratio. ^(b)^ Acryloid^®^ F-10 is n-butyl methacrylate.

**Table 7:** Polymers as adhesives for artworks in cultural heritage.

Polymer	Artwork to Be Glued	Advantages	Limitations/Drawbacks	References
Acrylic polymers, e.g., Paraloid^®^ B72, Paraloid^®^ B67 ^(a)^	- Adhesion of flaking paint layers - Bonding of paper, parchment - Bonding of canvas fragments - Fixing decorative elements on historic objects	- Good adhesion to a wide range of substrates - Optical transparency - Excellent long-term stability - Resistance to yellowing - Solubility in organic solvents allows for reversibility	- Sensitive to environmental factors - Low mechanical strength - Careful application to avoid surface gloss changes	[114,115,116,117]
PVAC, e.g., Vinavil^®^ and Mowilith^®^	- Wood - Textile - Bonding of composite materials and porous substrates	- Good flexibility - Easy to apply - Low cost - Widely available	- Low chemical stability - Yellowing over time - Limited reversibility	[26]
Epoxy resins, e.g., Araldite^®^ and EpoFix^®^	- Structural repairs of ceramics, stone sculptures, and architectural elements - Adhesive for high-stress areas requiring long-term stability	- High mechanical strength and excellent gap-filling ability - Chemical resistance - Durability - Versatility in bonding diverse substrates	- Limited reversibility - Potential color change or yellowing over time	[115,118]
Cellulose derivatives, e.g., MC and hydroxypropyl cellulose (HPC)	- Stabilization of fragile paper and textiles - Temporary adhesives for manipulation during (a) restoration	- Biocompatible and reversible - Excellent for delicate artefacts - Minimal impact on color and texture	- Low mechanical strength - Sensitive to microbial attack - Limited long-term durability in high-humidity environments	[27,119]

^(a)^ Paraloid B67 is poly(isobutyl methacrylate).

**Table 9:** Polymers for the surface protection of metals in cultural heritage.

Polymer	Artwork to Be Coated	Results	Advantages	Disadvantages	Reference
Paraloid^®^ B72	- Copper alloys - Bronze - Iron	- Good adhesion - Corrosion protection	- Transparent, - Widely accepted in conservation practice	- Limited long-term outdoor corrosion resistance - Periodic renewal is required	[139]
Paraloid^®^ B48N	Metal surfaces	Highly abrasion- resistant coating	- Better abrasion resistance compared to Paraloid^®^ B72, broadly similar protection	Aging	[139]
Incralac^®^, acrylic formulation with benzotriazole (BTA)	Outdoor bronze/copper alloy monuments	- Protective film - Corrosion inhibition	- Good corrosion resistance - Widely used for outdoor copper alloys - Expected service 3–5 years outdoors	- BTA is toxic - Solubility issues	[139]
Epoxy resins	Bonding and structural reinforcement of bronze artefacts	Very strong adhesion and mechanical support for fractured objects	High toughness and adhesion for structural repair	Poor reversibility, aging, discoloration or delamination over time	[140]
Polyethylene waxes	Ferrous and copper alloys	Better performance than natural waxes	Visual finish close to natural appearance	- Outdoor performance can fail - Limited corrosion protection	[139]
Chitosan-based coatings	Bronze/copper alloys	- Good corrosion barrier - It can incorporate inhibitors like BTA for controlled release	- Eco-friendly - Transparent and adherent	- Novel; long-term effectiveness and standardization still under research	[121]

**Table 10:** Polymers for the surface protection of polychrome and painted surfaces in cultural heritage.

Polymer	Artwork to Be Coated	Results	Advantages	Disadvantages	References
Paraloid^®^ B72 and B67	- Panel paintings - Murals - Polychrome sculpture	Transparent protective film	- Good transparency -Good protection - Reversible with solvents - Widely used in conservation	- Tend to undergo photo-oxidation, yellowing, embrittlement - Solubility changes over time - It may trap salts beneath film, leading to degradation and cracking	[121,141]
PVAC	Picture varnishes	Transparent protective film	- Historically important - Good adhesion to many substrates	- Attracts dirt - Poor leveling - It can hydrolyze releasing acetic acid, harmful to paint and substrate	[141]
Funori ^(a)^	- Wall paintings - Polychrome surfaces with friable layers	- Preserving matt surface - Good surface wetting	- Non-toxic - Matt drying - UV stability - Limited biological growth	- Hygroscopic - It can gel in presence of salts - It may reduce vapor permeability if overapplied	[142,143]
Gelatin and proteins	Cracked paint layers	- Good initial adhesion - Reversible with water	Traditional material familiar to historic practice	- Biological susceptibility - Yellowing, embrittlement degradation - Controlled application	[142]
HPC and MC	Delicate painted surfaces	- Transparent and stable films	- Reversible - Good compatibility	- It can attract dirt - Less effective for strong mechanical protection - Water-sensitive	[144]

*^(^*^a)^ Funori is a polysaccharide extracted from red seaweeds of the genus Gloiopeltis [145].

**Table 11:** Polymers for the surface protection of ceramics and terracotta in cultural heritage.

Polymer	Artwork to Be Coated	Results	Advantages	Disadvantages	Reference
Paraloid^®^ B72	- Ceramic tiles and pottery - Friable terracotta surfaces	- Hydrophobicity - Reduced water absorption	- Good transparency - Relatively reversible with solvents - Widely used in conservation	- Long-term durability concerns - Sensitivity to UV aging	[146]
Nano-Paraloid^®^ B72	- Archaeological pottery - Ceramics	Homogeneous coating	High mechanical reinforcement	- Research stage - Needs specialist formulation	[147]
Silicon-based coatings	- Outdoor terracotta artworks - Architectural ceramics	- Protecting film in artificial aging tests - Limited water absorption	- Effective water barrier - Durability for outdoor use	Careful application	[148]
Alkoxysilanes	Damaged pottery and ceramics	- Increased compressive strength - Hydrophobicity - Salt resistance - Improved mechanical properties	- Enhanced performance - Good vapor permeability - Limited color change	- Requires chemical expertise - Application protocols sensitive	[149]
HPC	Ceramic artefacts	- Hydrophobicity - High mechanical properties	Eco-friendly polymer	- Research stage - Needs standardized protocols for heritage practice	[150]

**Table 12:** Polymers for the surface protection of wooden objects in cultural heritage.

Polymer	Artwork to Be Coated	Results	Advantages	Disadvantages	Reference
Acrilic polymers, e.g., Paraloid^®^ B72, B67, B44	Wooden artefacts	Moisture resistance	- Transparent - Reversible in organic solvents - Widely used in conservation practice	- Degradation over time due to UV and microbes - It may alter appearance when overapplied	[152]
Acrylic polymer/SiO_2_ nanocomposites	Historic wooden artefacts	- Water repellence - UV resistance - Durability	- Multifunctional protection - Good transparency	- Complex synthesis - High cost	[153]
AgNP-containing acrylic/ siloxane coatings	Wooden heritage artefacts in museums and historic buildings	Inhibition of fungal and bacterial growth	Strong antimicrobial activity	- High cost - Nanoparticle release	[152]
Epoxy resins	Degraded wooden architectural	High bond strength	Good protection	- Not reversible - UV sensitivity - Heat generated during cure may damage wood	[155]
TiO_2_ nanoparticles	Outdoor wooden artefacts	- UV shielding - Self-cleaning properties	- Photocatalytic activity - Reduced surface degradation	-	[156]
ZnO nanoparticles	Wooden artefacts exposed to biological attack	- Antifungal protection - UV resistance	Antimicrobial activity	Possible nanoparticle aggregation and aesthetic changes	[157]

**Table 13:** Natural and synthetic polymers employed for the consolidation, protection, and stabilization of cellulosic materials in cultural heritage.

Polymer	Artefact	Purpose	Advantages	Disadvantages	Reference
Chitosan	- Paper - Cellulosic artefacts	- Consolidation -Antimicrobial protection	- Biocompatible - Biodegradable - Antimicrobial properties	- Hygroscopic - Possible changes in surface properties	[163]
Gelatin	Paper	- Consolidation - Surface sizing	Good adhesion to fibers	- Sensitive to humidity - Biological vulnerability	[164]
Carboxymethyl cellulose	- Paper - Textiles	- Consolidation - Sizing - Dimensional stabilization	- Good affinity - Water-soluble - Reversible	- Water sensitivity - Limited mechanical reinforcement	[165]
Cellulose nitrate	- Paper - Wood	- Protective coating - Adhesive	- Strong adhesion - Fast drying - Historical use	- Severe long-term instability - Yellowing - Embrittlement	[166]

**Table 14:** Representative case studies on the conservation of cellulosic artefacts.

Case Study	Artefact	Main Degradation Issues	Polymers Used	Conservation Strategy	Outcome	References
Japanese washi prints ^(a)^	Prints and scrolls	- Cellulose depolymerization - Oxidation induced by hydrolysis and UV light	- Kozo tissues ^(b)^ - Wheat starch paste - MC	- Reinforcement - Preventive conservation of mechanical and optical properties	- High compatibility - Reversibility	[171]
Leonardo da Vinci’s Codex and related notebooks	Manuscripts	- Acid-induced decomposition of cellulose - Mechanical fragility	- Kozo washi papers - Wheat starch paste - MC - PVA	- Deacidification - Mechanical reinforcement	Stabilization of cellulose	[168,172]
Dutch National Archives	Manuscripts	- Ink corrosion - Embrittlement - Perforation	Phytate- calcium-based treatment	- Iron chelation - Reduced oxidation - Improved mechanical stability		[173,174]
British Library collections	Iron gall ink documents	- Ink corrosion - Embrittlement	Japanese tissues applied with starch-based adhesives	- Deacidification - Reinforcement	Effective large-scale stabilization	[170]
Historical maps	Cartographic documents	- Ink corrosion - Localized degradation	- Japanese tissues applied with starch-based adhesives - Local phytate treatment	- Deacidification - Reinforcement	- Preservation of readability – Dimensional stability	[175]
Notarial documents	Legal/archival records	- Depolymerization - Ink corrosion - Alum-related acidity	Local phytate treatment	Reinforcement	- Improved mechanical stability - Scalable conservation protocols	[169]

^(a)^ Traditional Japanese washi paper, produced from kozo, mitsumata, or gampi fibers [143,176,177,178], is a natural cellulose-based material characterized by long fibers, high crystallinity, and remarkable mechanical strength. ^(b)^ Kozo, obtained from the inner bark of the paper mulberry (Broussonetia papyrifera), is the most widely used fiber in conservation-grade washi because of its exceptional tensile strength, flexibility, dimensional stability, and long-term durability. Washi is typically applied using reversible, cellulose-compatible natural polymer adhesives, such as wheat starch paste or methyl cellulose (MC).

**Table 15:** Critical analysis of conservation treatments applied to waterlogged ships, evaluating polymer degradation according to wood type, treatment methodology, and contextual observations.

Ship	Date	Location	Wood	Degradation Level	Polymer	References
Oseberg ships	~820 AD	Norway	Oak/Pine	Cellulose depolymerization	- PEG - Polysaccharide mixtures	[191]
Nydam boat	~320 AD	Denmark	Oak	Cellulose depolymerization	PEG	[192,193]
Hjortspring boat	350 BC	Denmark	Oak	- Cellulose depolymerization - Partial hemicellulose loss - Thin, fragile cell walls	Low MW PEG	[194]
Yenikapı shipwrecks	5th–11th centuries	Turkey	Oak/mixed species	- Cellulose depolymerization - Highly degraded, porous timbers	PEG	[195]
Bremen Cog	~1380	Germany	Oak	- Cellulose depolymerization - High porosity	Various MW PEG	[196]
Newport ship	1450s	UK	Oak	- Cellulose and hemicellulose depolymerization - Collapsed cell walls	PEG 200/PEG 4000	[197]
Mary Rose	1545	UK	Oak	Cellulose, hemicellulose and lignin depolymerization	PEG 200/PEG 2000	[198]
Vasa ship	1628	Sweden	Oak	- Cellulose and hemicellulose depolymerization - Oxidative degradation catalyzed by residual sulfur and iron traces	PEG 400/PEG 4000	[199,200,201]

**Table 16:** Case studies of wooden musical instruments: wood type, degradation, and acoustic outcomes.

Instrument	Date	Wood	Degradation Level	Acoustic/Sound Outcomes	References
Flemish harpsichord	1600s	- Soundboard: spruce - Frame: oak	Soundboard flattening due to yearly humidity cycles	Partial restoration of crown and vibrational response	[207]
Stradivarius violin	1700s	- Top: spruce - Back and side: maple	- Natural long-term aging - Hemicellulose reduction - Slight lignin oxidation	- Enhanced resonance - Sustain, harmonic richness - Improved projection	[202,203,204]
Guarneri violin	1700s	- Soundboard: spruce - Frame: maple	- Natural long-term aging - Hemicellulose reduction - Slight lignin oxidation	- Increased vibrational response - Increased tonal clarity	[202,203,204]
Cathedral pipe organ	18th century	- Pipes: oak - Bellows: pine	Mold growth, cracking from poor airflow and heating	- Restored resonance - Structural stabilization	[208]
Vuillaume-school cello	19th century	- Top: spruce - Back and side: maple	- Bass-bar weakening due to string pressure - Mild fungal activity	- Acoustic balance restored - Enhanced power	[204]
1890 Steinway model B piano	Late 19th century	- Soundboard: spruce - Bridges: maple	- Soundboard cracks - Bridge detachment due to dry storage	- Acoustic recovery - Restored warmth and resonance	[209]
Martin D-28 guitar	Pre-war 1930s	- Top: Sitka Spruce - Back and side: Brazilian Rosewood	- Resin crystallization - Moisture loss - Density changes	- Richer bass response - Improved sustain - Enhanced tonal complexity	[204,210]
Grenadilla clarinet	20th century	African Blackwood	Barrel cracks due to low humidity	- Internal reinforcement - Structural stability - Tonal quality maintained	[174]

**Table 17:** Artists and designers using polymeric materials: decomposition mechanisms, analytical markers, and conservation issues.

Artist/Designer	Polymer	Main Decomposition Mechanisms	Macroscopic Effects
Alberto Burri	Mixed polymers	Thermal oxidation	Cracking
Anish Kapoor	Synthetic polymers	Surface decomposition	Color shift
César Baldaccini	PU, polyester	Oxidation	Cracking
Eva Hesse	Latex, PVC	- Oxidation - Plasticizer migration	Collapse
Guzzini collection	PMMA, PC, melamine–formaldehyde resins	- Hydrolysis - Photo-oxidation	- Yellowing - Cracking
Jesús Rafael Soto	PVC, PA6	Creep	Deformation
Joe Colombo	PVC, ABS	Differential degradation	Warping
Kartell collection	PMMA, ABS, PC	Photo-oxidation	Loss of transparency
Naum Gabo	Cellulose acetate	Deacetylation	Embrittlement
Niki de Saint Phalle	Polyester	Photo-oxidation	Cracking
Olivier Mourgue	PUF	- Hydrolysis - Oxidation	Elasticity loss
Rachel Whiteread	Epoxy, polyester	Photo-oxidation	Yellowing
Tony Cragg	Mixed polymers	- Oxidation - Plasticizer migration	Heterogeneous decay

## Data Availability

The raw/processed data required to reproduce the above findings cannot be shared at this time as the data also form part of an ongoing study.

## Abbreviations

The following abbreviations are used in this manuscript:

**Acronym**	**Name**
BA	Butyl acrylate
BTA	Benzotriazole
DN	Double-network hydrogels
EDTA	Ethylenediaminetetraacetic acid
EMA	Ethyl methacrylate
FT-IR	Fourier-transform infrared spectroscopy
HPMC	Hydroxypropyl methylcellulose
HPC	Hydroxypropyl cellulose
IPN	Interpenetrating network
LCST	Lower critical solution temperature
MA	Methyl acrylate
MC	Methylcellulose
MMA	Methyl methacrylate
NMR	Nuclear magnetic resonance spectroscopy
O/W	Oil-in-water
PAA	Poly(amidoamine)
PDMS	Poly(dimethylsiloxane)
PE	Poly(ethylene)
PEG	Poly(ethylene glycol)
PEO	Poly(ethylene oxide)
PFPE	Perfluoropolyether
pHEMA	Poly(2-hydroxyethyl methacrylate)
PMMA	Poly(methyl methacrylate)
PNIPAM	poly(N-isopropylacrylamide)
PPO	Poly(phenylene oxide)
PU	Polyurethane
PUF	Polyurethane foam
PVA	Poly(vinyl alcohol)
PVAC	Poly(vinyl acetate)
PVB	Poly(vinyl butyral)
PVC	Poly(vinyl chloride)
PVP	Poly(vinyl pyrrolidone)
Semi-IPN	Semi-interpenetrating network
SDG	Sustainable development goal
3D	Three-dimensional
UNESCO	United nations educational, scientific and cultural organization
UV	Ultraviolet
VOC	Volatile organic compound
